# Mathematical model of hypoxia and tumor signaling interplay reveals the importance of hypoxia and cell-to-cell variability in tumor growth inhibition

**DOI:** 10.1186/s12859-019-3098-5

**Published:** 2019-10-21

**Authors:** Emile P. Chen, Roy S. Song, Xueer Chen

**Affiliations:** 1 Computational Sciences, GlaxoSmithKline, Collegeville, PA 19426 USA; 2 Department of Biomedical Informatics, University of Pittsburgh, Pittsburgh, PA 15206-3701 USA

**Keywords:** Mathematical tumor model, Hypoxia tumor signaling, Tumor signaling, Tumor growth model

## Abstract

**Background:**

Human tumor is a complex tissue with multiple heterogeneous hypoxic regions and significant cell-to-cell variability. Due to the complexity of the disease, the explanation of why anticancer therapies fail cannot be attributed to intrinsic or acquired drug resistance alone. Furthermore, there are inconsistent reports of hypoxia-induced kinase activities in different cancer cell-lines, where increase, decreases, or no change has been observed. Thus, we asked, why are there widely contrasting results in kinase activity under hypoxia in different cancer cell-lines and how does hypoxia play a role in anti-cancer drug sensitivity?

**Results:**

We took a modeling approach to address these questions by analyzing the model simulation to explain why hypoxia driven signals can have dissimilar impact on tumor growth and alter the efficacy of anti-cancer drugs. Repeated simulations with varying concentrations of biomolecules followed by decision tree analysis reveal that the highly differential effects among heterogeneous subpopulation of tumor cells could be governed by varying concentrations of just a few key biomolecules. These biomolecules include activated serine/threonine-specific protein kinases (pRAF), mitogen-activated protein kinase kinase (pMEK), protein kinase B (pAkt), or phosphoinositide-4,5-bisphosphate 3-kinase (pPI3K). Additionally, the ratio of activated extracellular signal-regulated kinases (pERK) or pAkt to its respective total was a key factor in determining the sensitivity of pERK or pAkt to hypoxia.

**Conclusion:**

This work offers a mechanistic insight into how hypoxia can affect the efficacy of anti-cancer drug that targets tumor signaling and provides a framework to identify the types of tumor cells that are either sensitive or resistant to anti-cancer therapy.

## Introduction

It is well appreciated that many cancers result from dysregulation of multiple signaling pathways that alters fundamental processes of cell proliferation, growth and survival [1]. Such insights have led to the successful development of anti-cancer drugs that target these pathways [2, 3]. In the last two decades, advances in systems-based modeling of cell signaling networks have improved our ability to predict the effectiveness of drugs targeting various points of these pathways [4]. Due to the extensive cross-talk and compensatory feedback interactions within the network, such predictions would be otherwise difficult to make without the aid of advanced mathematical modeling techniques.

However, the effectiveness of anti-cancer drugs cannot be adequately predicted without also considering how hypoxia alters the signaling network dynamics [5]. It has been estimated that many solid tumors contain hypoxic regions due to poor blood flow resulting from aberrant development of new blood vessels [6]. Tumor cells undergo multiple adaptive changes to enable survival and proliferation under a reduced oxygen environment, which could impact drug efficacy [7]. Among these changes, activation of the hypoxia-inducible-factor (HIF) signaling pathway leads to increased production of several proteins. For example, vascular endothelia growth factor (VEGF) [8] has been shown to mitigate the hypoxic conditions in the tumor microenvironment by inducing angiogenesis. Also, VEGF binding to the VEGF receptor (VEGFR) can lead to ERK and Akt activation [9], enhancing proliferation and survival of tumor cells. However, under severe hypoxic conditions, the depletion of adenosine triphosphate (ATP) disrupts reactions that require ATP-dependent phosphorylation that are critical to ERK and Akt activation [10]. To add to the complexity, it is becoming clear that significant cell-to-cell variability of biomolecule levels including proteins and phospholipids, occur within the tumor cell population [11–13]. These subpopulations of tumor cells with different range of protein expression have been reported to exhibit categorical differences in their response to external stimuli [13]. For example, different cancer cell-lines have contrasting Akt activity under hypoxic conditions; There are reports that Akt phosphorylation increases or remains unchanged under hypoxia [14–16]. However, the mechanism that drives differences in hypoxia-induced Akt activity is unclear. Thus, we asked two questions. Can we build a model that can elucidate the underlying mechanism of hypoxia-induced cancer cell growth and explain why such variability between tumor cell-types exist? Can we increase our ability to design more effective cancer therapy with further understanding of the prominent role of hypoxia and heterogenicity on the growing tumor population? We address these questions by conducting a simulation study to explore how hypoxia-induced shifts in tumor signaling dynamics impact tumor growth and drug response.

We developed our model by integrating individual published models that reflect the intracellular tumor signaling pathway and the hypoxia-induced signaling pathway by using VEGF mRNA output from one model as input to the next model to drive VEGF production. Additionally, ATP-dependent phosphorylation was added to all phosphorylation reactions in the tumor signaling pathway to incorporate hypoxia induced ATP depletion. The combined model (illustrated in Fig. 7) and how the individual model components are integrated are detailed in the method section. We qualitatively validated our model by being able to reproduce diverging hypoxia-induced kinase activity observed in literature. We applied the model to explain why different subpopulation of tumor cells could respond differently from the same stimuli. To do this, we performed a global sensitivity analysis followed by decision tree modeling to determine patterns in biomolecular concentrations that result in diverging network behavior. Since heterogeneity is a hallmark of cancer, our ability to predict how different subpopulations of tumor cells would respond differently to drug treatments will better equip us to derive more meaningful predictions to guide drug discovery and development.

## Results

All results herein are simulation generated from our model. Because our model is built upon previously published models, each individual model was tested to ensure that simulated output from the original publication could be reproduced before moving further. We developed our model by interfacing the output of one model as input to the next model. Moreover, the tumor signaling model, originally developed for normal oxygen level, was modified to account for hypoxia-induced changes in the VEGF synthesis rate and phosphorylation rates of several signaling proteins. As detailed in Eqs. 2, 3 and 4 in the method section, these changes were introduced to ensure the same behavior of the combined model under normoxic condition.

Exploratory analysis reveals that the hypoxia or drug induced changes in pAkt and pERK levels are highly dependent on biomolecules concentration. To explore this dependency, model predicted changes of pAkt and pERK in response to step changes in O_2_ level and/or drug concentration were evaluated using 20,000 random combinations of protein and/or phospholipid levels. All subsequent mentioning of “responses” is model-predicted, “cases” refer to simulation cases using different initial biomolecule levels and can be considered as “cells”.

### Steady state concentration distribution of biomolecules

**Fig. 1:**
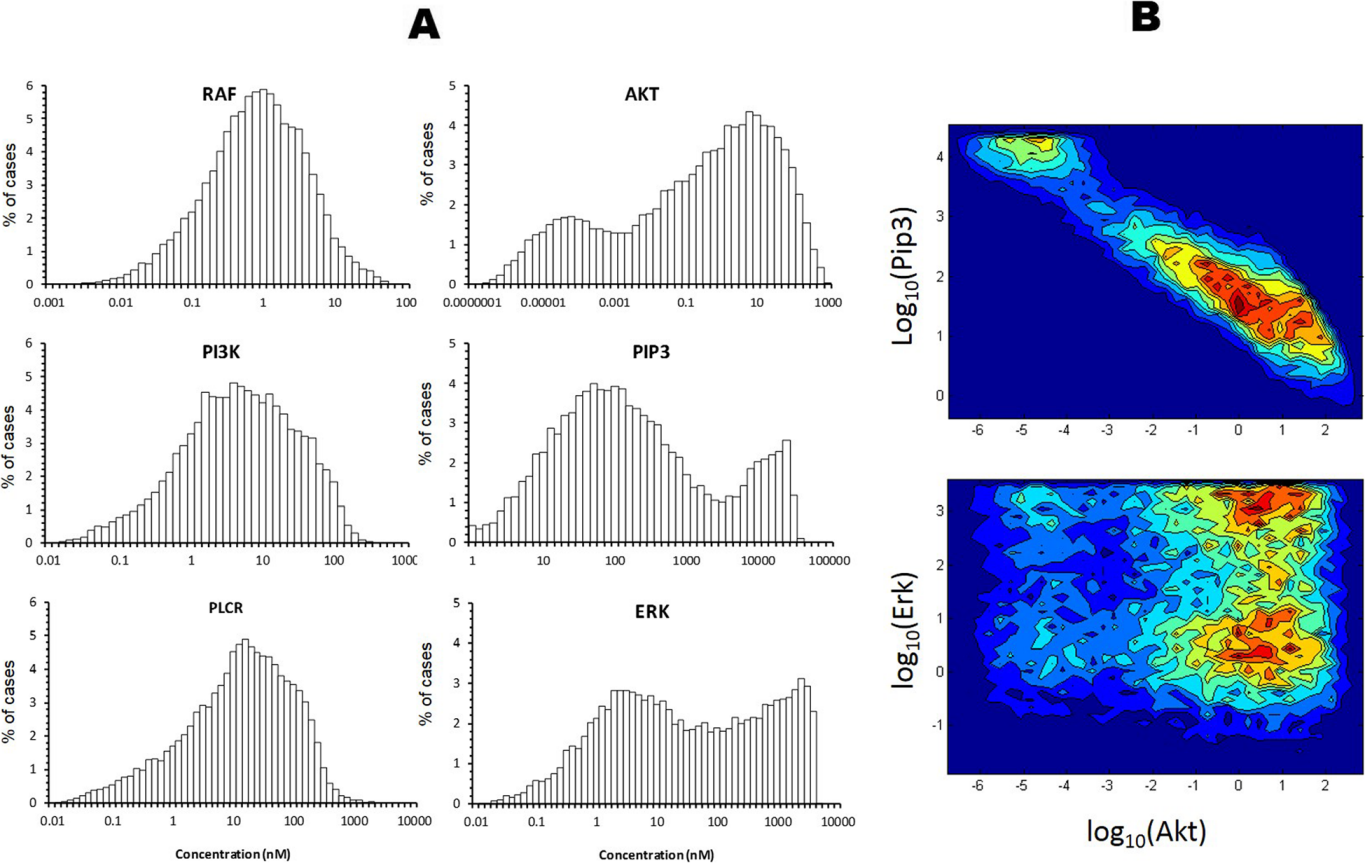
Steady state biomolecule concentration exhibits normal and bimodal distribution. **a** Histograms of selected biomolecule levels at steady-state after repeated simulation (*n* = 20,000) using randomly selected initial concentration levels. Most biomolecules exhibit normal or near normal distributions. Skewed distributions were seen in six of them: PI3K, pERK, PIP3_Akt (the complex of PIP3 and Akt), pPIP3_Akt, MEK, PLCR (phosphorylated phospholipase Cγ1). Seven biomolecules exhibit bimodal distribution: Akt, ERK, PIP3 (phosphatidylinositol-4,5-bisphosphate 3-kinase), Rafi (inhibited RAF), pRsk (phosphorylated ribosomal s6 kinase), Rsk, Pip3_PDK (the complex of PIP3 and phosphoinositide dependent protein kinase). **b** Examples of two-dimensional histograms showing correlation of biomolecule concentrations clustering into distinct subpopulations

Although biomolecule concentrations were initially generated as a uniform random distribution, the final steady-state concentrations were not uniformly distributed (6 examples shown in Fig. 1a). Most (> 85%) of biomolecules exhibited normal or nearly normal distributions (e.g. RAF), while six showed skewed distributions and seven showed bimodal distribution.

As would be expected, the VEGF and various forms of the receptor-growth factor complex are positively correlated. VEGFR is also negatively correlated with pAkt. Distinct clusters of biomolecule concentrations were also observed from the simulation as subpopulations. For example, the concentrations of Akt and PIP3 cluster into two distinct subpopulations (Fig. 1b, top), whereas the concentration of Akt and ERK clustered into 4 subpopulations (Fig. 1b, bottom).

### Hypoxia-induced effect on mRNA and VEGF productions

**Fig. 2:**
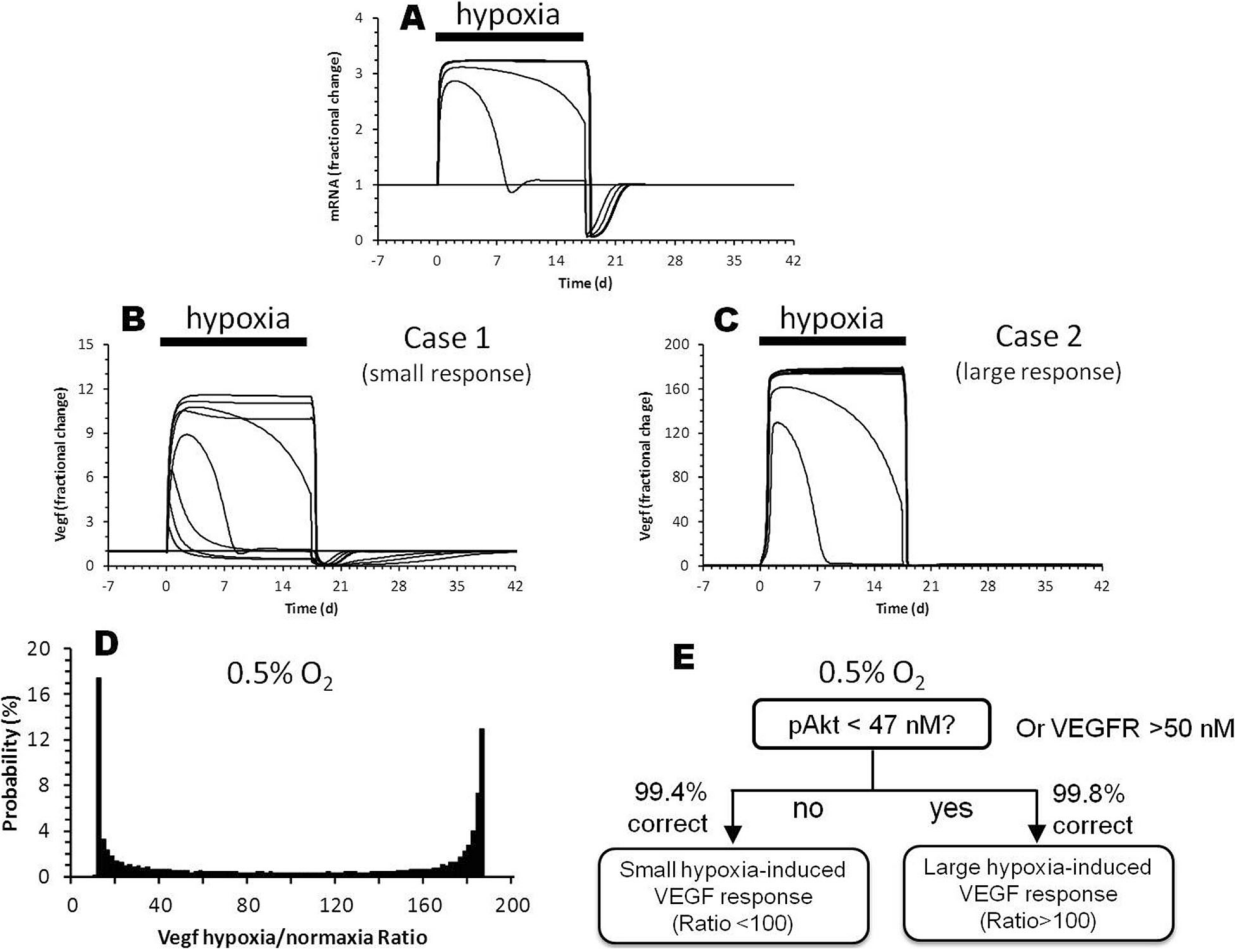
Increasing hypoxia amplifies VEGF production. **a** Each solid line represents the response of mRNA to step reduction of O_2_ from normoxia (100% O_2_) to a reduced O_2_ level. The response is normalized to the mRNA baseline level at normoxia (baseline = 1). **b** and **c** Two simulation cases demonstrating that, by using different initial levels of biomolecules, either a “small” or “large” change in VEGF level (maximal changes of either 12-fold or 160-fold) can be elicited in response to the same mRNA response to hypoxia depicted in (**a**). Note the difference in the scale of y-axis. **d** Histogram depicting the distribution of VEGF response magnitude from 20,000 simulations in response to one step changes of O_2_ level from 100 to 0.5%. **e** Decision tree showing that pAkt level is the sole factor dictating the magnitude to “large” (> 100) versus “small” (< 100) VEGF response

Figure 2a shows the simulated effect of prolonged change in O_2_ (0.001–100%, with 100% defined as 21% O_2_ under normoxia) on VEGF mRNA transcription. Both VEGF and its mRNA increase transiently following mild hypoxia. As hypoxia becomes more severe, the VEGF response is amplified and more sustained. Interestingly, the magnitude of steady-state VEGF response depends significantly on the concentrations of specific biomolecules when simulated with the same degree of hypoxia (two cases in Fig. 2c) that can be clustered into “big” versus “small” increases in mRNA transcription (Fig. 2d).

Decision tree analysis revealed that pAkt level is the most principal factor that determines whether the model results in a big or small hypoxia–induced VEGF response (Fig. 2e). At the 0.5% O_2_ hypoxic level, cells with pAkt greater than 47 nM mostly produce a substantial change, while cells with pAkt < 47 nM mostly produce a small change. However, the impact of pAkt on VEGF response diminishes as severity of hypoxia decreases.

### Effect of hypoxia on ERK and Akt activation

Our model shows that, although pERK and pAkt generally decrease with increasing hypoxia, both the magnitude and direction of the hypoxia-induced changes are highly dependent on the concentration of those biomolecules. For some cases, the model predicts that the levels of pERK and pAkt will remain unchanged or even increase during severe hypoxia.

### Hypoxia and pERK

**Fig. 3:**
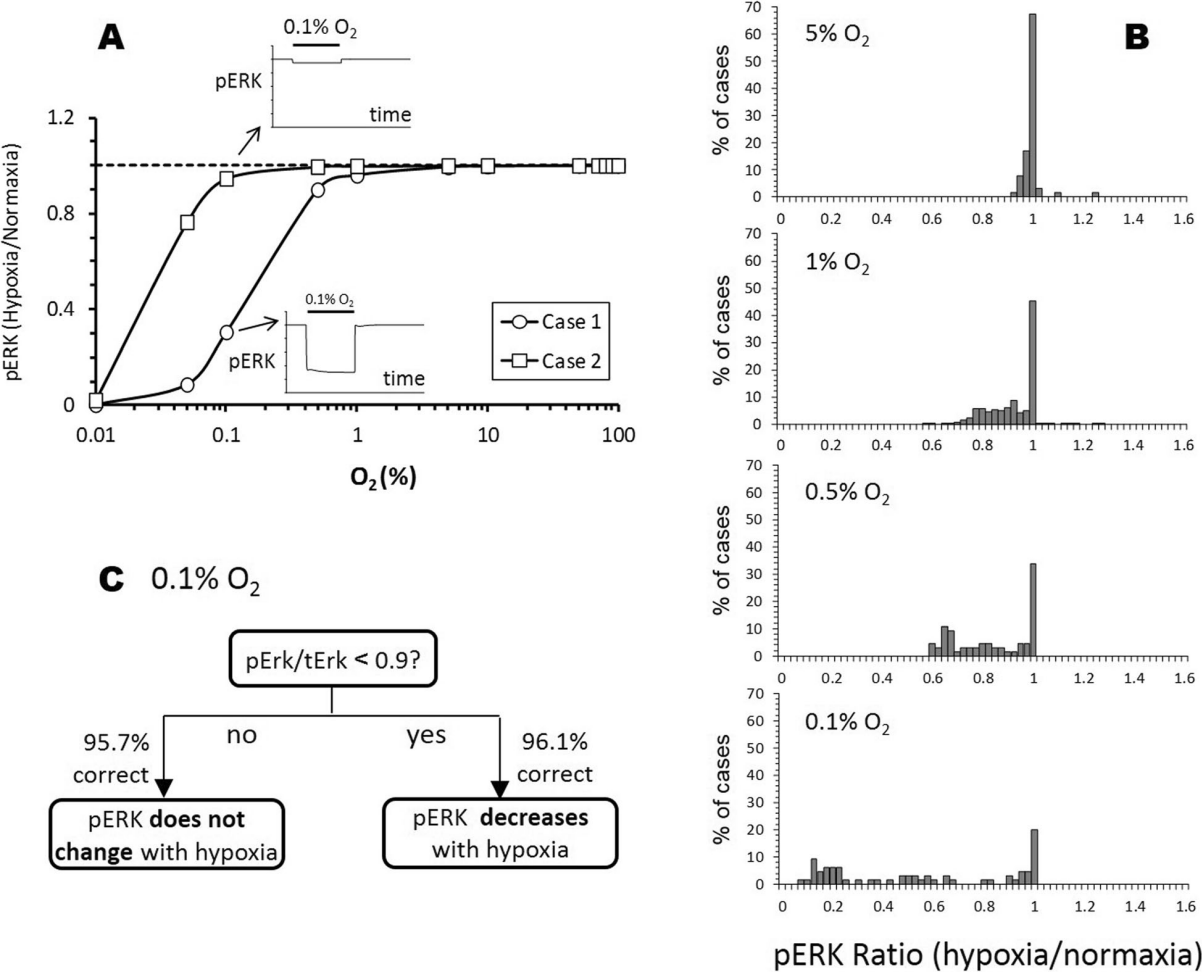
Hypoxia induces a decrease or no change in ERK activation. **a** Two simulation cases showing differential sensitivity in steady-state pERK response to step reduction in O_2_ levels of various severity (normoxia = 100%). The time course for pERK responses to 0.1% O_2_ is illustrated in the insert, where x-axis is time and y-axis is the level of phosphorylated ERK. The solid bar above the line trace indicates when 0.1% O_2_ was applied in the simulation. **b** Histograms summarizing the distribution of pERK responses from all 20,000 simulation cases at various degree of hypoxia. **c** Decision tree analysis was used to identify which biomolecule levels are most important in determining the sensitivity of pERK to hypoxia by categorizing the simulation cases into “no change” or “decreased (within ±10% change or > 10% reduction from the normoxia level). The resultant decision tree showing that pERK/tERK ratio is the primary factor deciding if pERK is sensitive to hypoxia (0.1% O_2_) or not

Our model simulation show that hypoxia can decrease or have minimal effect on pERK levels (Fig. 3a). Our model predicts that the 5% O_2_ level has minimal effect on ERK activity. About 96% of cases show no change in pERK (Fig. 3b; hypoxia/normoxia pERK ratio between 0.9 and 1.1) when simulated at the 5% O_2_ hypoxic level. Our model also shows that as hypoxia level increases, ERK activation decreases. At the 0.5% O_2_ level, a multi-modal distribution was observed where 66% of cases with greater than 20% reduction in pERK fell into one cluster (hypoxia/normoxia pERK ratio < 0.8), while 28% with minimal change in pERK (hypoxia/normoxia pERK ratio between 0.9 and 1.1) fell into another cluster. Interestingly, the model predicts that even at a low O_2_ level of 0.5%, a subpopulation of tumor cells still exhibits minimal change in ERK activity.

Decision tree analysis (Fig. 3c) identified pERK/tERK, the ratio of phosphorylated ERK over total ERK, rather than the absolute levels of either pERK or ERK, as the single most important feature in determining the sensitivity of pERK to hypoxia. Cells with pERK/tERK ratio less than 0.9 at 0.1% O_2_ will most likely result in a decrease in pERK (defined as > 10% reduction in pERK). Cells with pERK/tERK ratio greater than 0.9, hypoxia will have a minimal or no effect on ERK activity.

### Hypoxia and pAkt

**Fig. 4:**
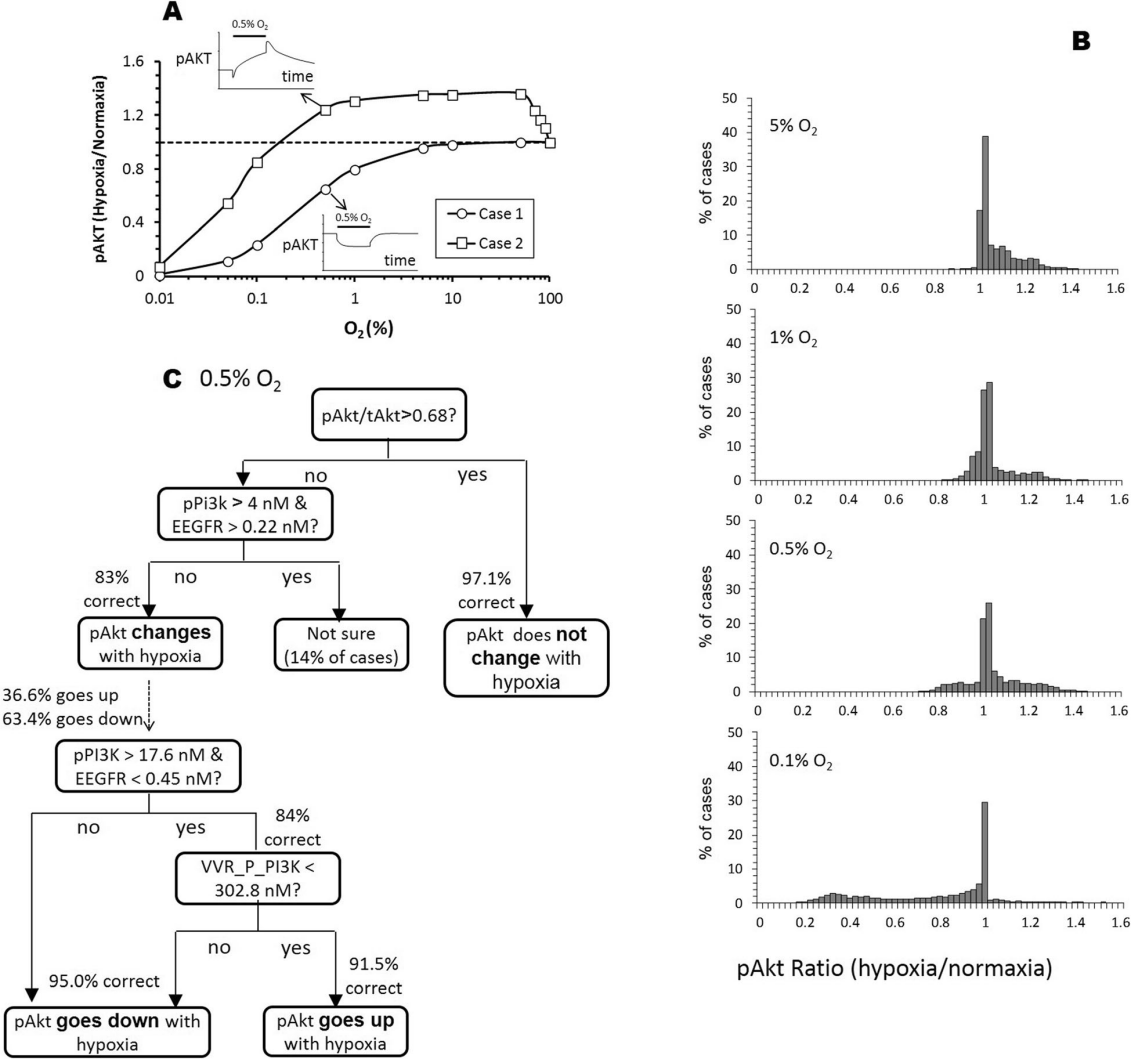
Hypoxia can induce an increase, decrease, or no change in Akt activity. **a** Two simulation cases showing differential steady-state pAkt responses to step reduction in O_2_ levels of various severity (normoxia = 100%). The time course for the pAkt responses to 0.5% O_2_ is illustrated in the insert, where x-axis is time and y-axis is the level of phosphorylated ERK. The solid bar above the line trace indicates when 0.5% O_2_ was applied in the simulation. **b** Histograms summarizing the distribution of pAkt responses from all 20,000 simulation cases at various degrees of hypoxia. **c** Decision tree showing that pAkt/tAkt ratio is the key factor deciding how pAkt response to hypoxia (0.5% O_2_), with pPI3K, EEGFR (the complex of EGF and its receptor) and VVR_P_PI3K (the complex of PI3K and the phosphorylated dimer of VVEGFR) also playing contributing roles

For Akt activity, we observed a more complicated hypoxia-induced response. In some cases, we saw a decrease in pAtk levels as hypoxic condition increases (Fig. 4a). In other cases, we noticed that pAkt levels increase under milder hypoxia, where only under severe hypoxic conditions did we finally see a decrease in pAkt levels (Fig. 4a). Deconvolution of the model revealed that such bimodal behavior results from the two opposing effects, the hypoxia-induced increase in VEGF response and the simultaneous ATP depletion. At mild to moderate hypoxic condition, there is minimal change in ATP concentration, yet the increase in HIF-1α signaling from VEGFR stimulation activates Akt that ultimately result in increased pAkt levels. At moderate hypoxic condition, VEGF production remains saturated, whereas the loss of ATP depletion becomes significant resulting in reduced pAkt level.

Because of this hypoxia-induced bimodal behavior, a tumor cell can either have elevated, suppressed, or unchanged pAkt at any given O_2_ level (Fig. 4b). At the 5% O_2_ level, hypoxia caused an increase in pAkt level in 76% of simulated cases, while having minimal or no effect on pAkt levels (defined as within ±10% change) in 24% of the cases. Interestingly, multi-modal distribution can clearly be seen at 0.5% O_2_, where the pAkt level can be elevated (22%), unchanged (67%), or suppressed (11%) by hypoxia. As O_2_ was further reduced to 0.1%, the hypoxia-induced increase in pAkt is lost (4%). Additionally, 47% of the cases with minimal or no change in the pAkt level remains and the number of cases with decreased pAkt level is increased to 41%.

To identify factors that drive the three types of hypoxia-induced responses in pAkt levels we performed a decision tree analysis in two stages using the cases simulated at 0.5% O_2_. The first tree (Fig. 4c, top tree) was built to distinguish between no change (within ±10% change from normoxia level) versus change (greater than 10% change in either direction). Then, a second tree (Fig. 4c, bottom tree) was built to further separate the cases from the change category into either pAkt increase (> 10% change) or pAkt decrease (<− 10% change).

Our decision tree analysis identified pAkt/tAkt, the ratio of phosphorylated Akt over total Akt, as the most important variable in distinguishing between no change versus change categories. For tumor cells with pAkt/tAkt > 0.68, hypoxia will most likely have no effect on pAkt levels (97.1% accuracy). Ratio of pAkt/tAkt < 0.68, does not automatically imply that pAkt will likely change with hypoxia (28.2% misclassification error). Applying the second decision tree node (pPI3K < 4 nM or EEGFR (complex of EGF and its receptor) < 0.22 nM) is required to further reduce the misclassification error from 28.2% down to 17%.

There is a report that the bimodal response of Akt activity occurs in culture and suggests that this behavior is dependent on EGF stimulation and correlates with PI3K levels [13]. In agreement with the above study, our decision tree analysis also implicates pPI3K and EGFR as the two most crucial factors that determine the bimodal distribution of pAKT response in tumors under hypoxia. Our decision tree predicts that for cells with pPI3K levels greater than 17.6 nM and EEGFR levels lower than 0.45 nM, hypoxia will probably cause pAkt to go up (84% accuracy). Otherwise, pAkt will mostly go down with hypoxia (96% accuracy). An additional decision mode, VVR_P_PI3K (complex of PI3K and phosphorylated dimer of VVEGFRP) < 308.2 nM, is needed to further refine the classification.

### Effect of hypoxia on drug sensitivity

We used the model to investigate how hypoxia can alter the efficacy of different potential therapies. We examined the effect of hypoxia on inhibitors that target phosphorylation of RAS, MEK or Akt phosphorylation. Additionally, we also explored if hypoxia affects drugs that inhibit VEGF binding to VEGFR and how this affects ERK and Akt activation. Despite the interconnectivity of Ras-Raf-MEK-ERK and Akt signaling pathways, the model predicts that the inhibition of RAF or MEK primarily affects ERK activation and not Akt under hypoxia. Instead, the model predicts that the inhibition of Akt phosphorylation or VEGF binding to VEGFR mostly affects Akt activation.

### Effect of RAF and MEK inhibition on ERK activation under hypoxia

**Fig. 5:**
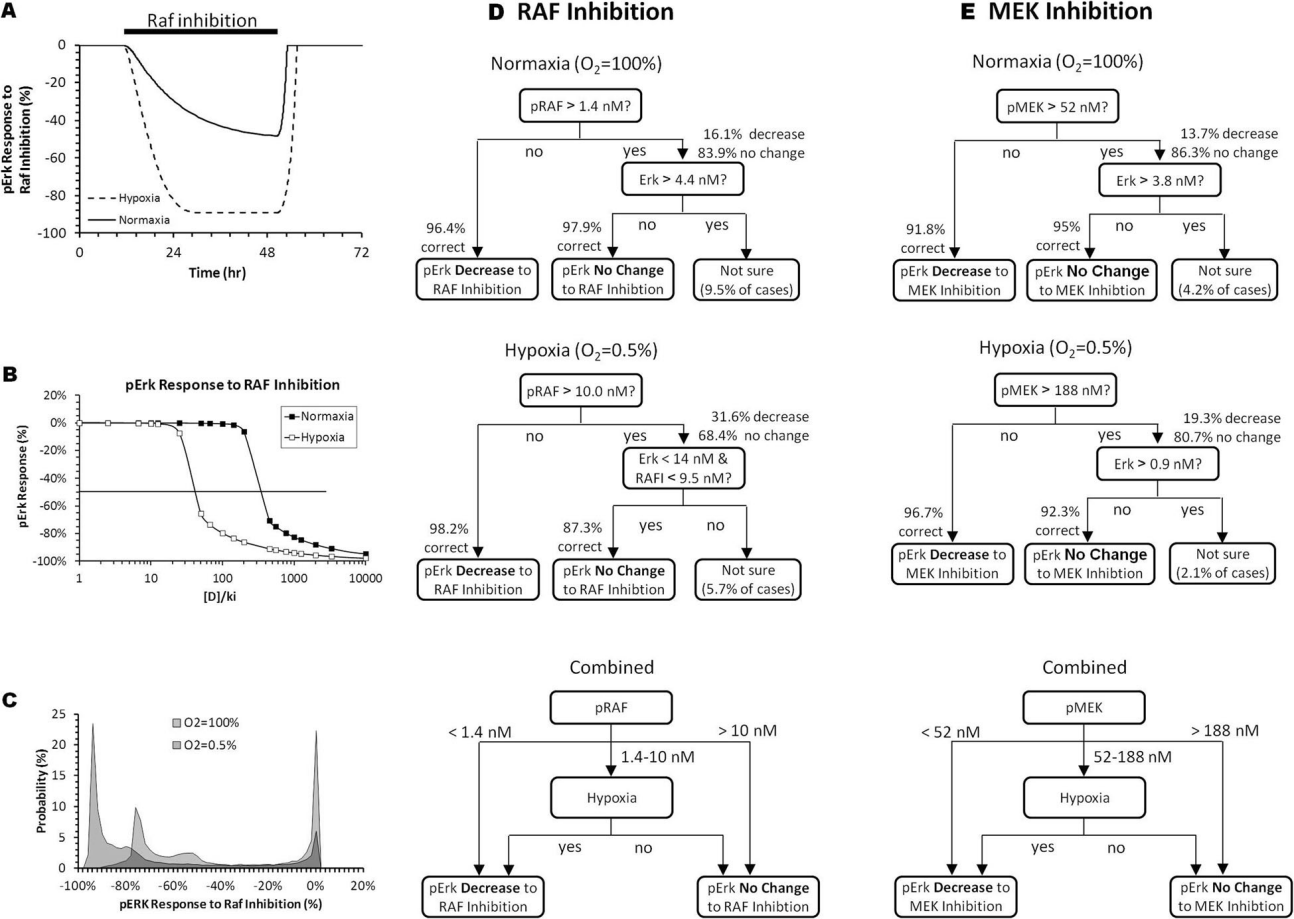
pRAF or pMEK concentration determines the level of pERK suppression by RAF or MEK inhibitors, respectively, under different hypoxic conditions. **a** One simulation case showing the pERK responses to inhibition of RAF phosphorylation under normoxia or sustained reduction of O_2_ from 100 to 0.5%. **b** Concentration response curves from one simulation case showing hypoxia increases the sensitivity of pERK reduction to inhibition of RAF phosphorylation. **c** Histograms summarizing the distribution of pERK responses to the same RAF inhibition from all 20,000 simulation cases under normoxia and hypoxia (0.5% O_2_). **d** and **e** Decision tree diagrams illustrating the key biomolecules dictating how pERK responses to inhibition of RAF or MEK phosphorylation. [D]/k_i_ = 100 and 2000 for inhibition of RAF and MEK phosphorylation, respectively

Figure 5a shows that in one specific case, the suppression of pERK levels by inhibition of RAF phosphorylation is enhanced under hypoxia. When tested across a range of drug concentrations (expressed as drug concentration to *K*_*i*_ ratios, *[D]/K*_*i*_), we observed that reducing the O_2_ level to 0.5% shifts pERK with 50% inhibition about 10-fold (Fig. 5b). When we repeated the simulation using different biomolecule concentrations, we observed that the same drug concentration (e.g. (*[D]/K*_*i*_ = 100) can either strongly inhibit pERK level (> 40% reduction in pERK) or become ineffective (< 10% reduction in pERK) (Fig. 5c). Along with hypoxia (0.5% O_2_, red histogram), this bimodal response distribution also occurred under normoxia (blue histogram). However, a larger proportion of cases had greater pERK reduction under hypoxia.

Decision tree analysis (Fig. 5d and e) revealed that the concentration of pRAF was the major contributor for the model sensitivity to RAF inhibitor, with minor contributions from ERK and RAFi levels. By combining the normoxia and hypoxia trees (Fig. 5d bottom), we saw that tumor cells with pRAF levels less than 1.4 nM are sensitive to RAF inhibition, whereas, tumor cells above 10 nM are insensitive to RAF inhibition regardless of O_2_ accessibility. Additionally, tumor cells with pRAF level that fall in-between 1.4 and 10 nM, that has accessibility to O_2_ (e.g. near the tumor surface with good vascularization) will be resistant to RAF inhibition. Yet, if these tumor cells are in hypoxic condition (e.g. cells near the tumor core with poor vascularization), they will be more sensitive to RAF inhibition.

Similarly, inhibition of MEK phosphorylation can also lead to a reduction in pERK levels and this effect is also more pronounced under hypoxia than under normoxia. Like RAF inhibition, not all cases are responsive to a given level of MEK inhibition, where the primary contributor of pERK bimodality is pMEK, with minor contributions from ERK (Fig. 5e). Tumor cells with pMEK level less than 52 nM are sensitive to MEK inhibition regardless of O_2_ accessibility and anything above pMEK levels of 188 nM are insensitive to MEK inhibition. Finally, for tumor cells with pMEK levels within the 52 and 188 nM range, the sensitivity depends on accessibility to O_2_.

### Inhibition of VEGF binding and Akt phosphorylation on Akt activation

**Fig. 6:**
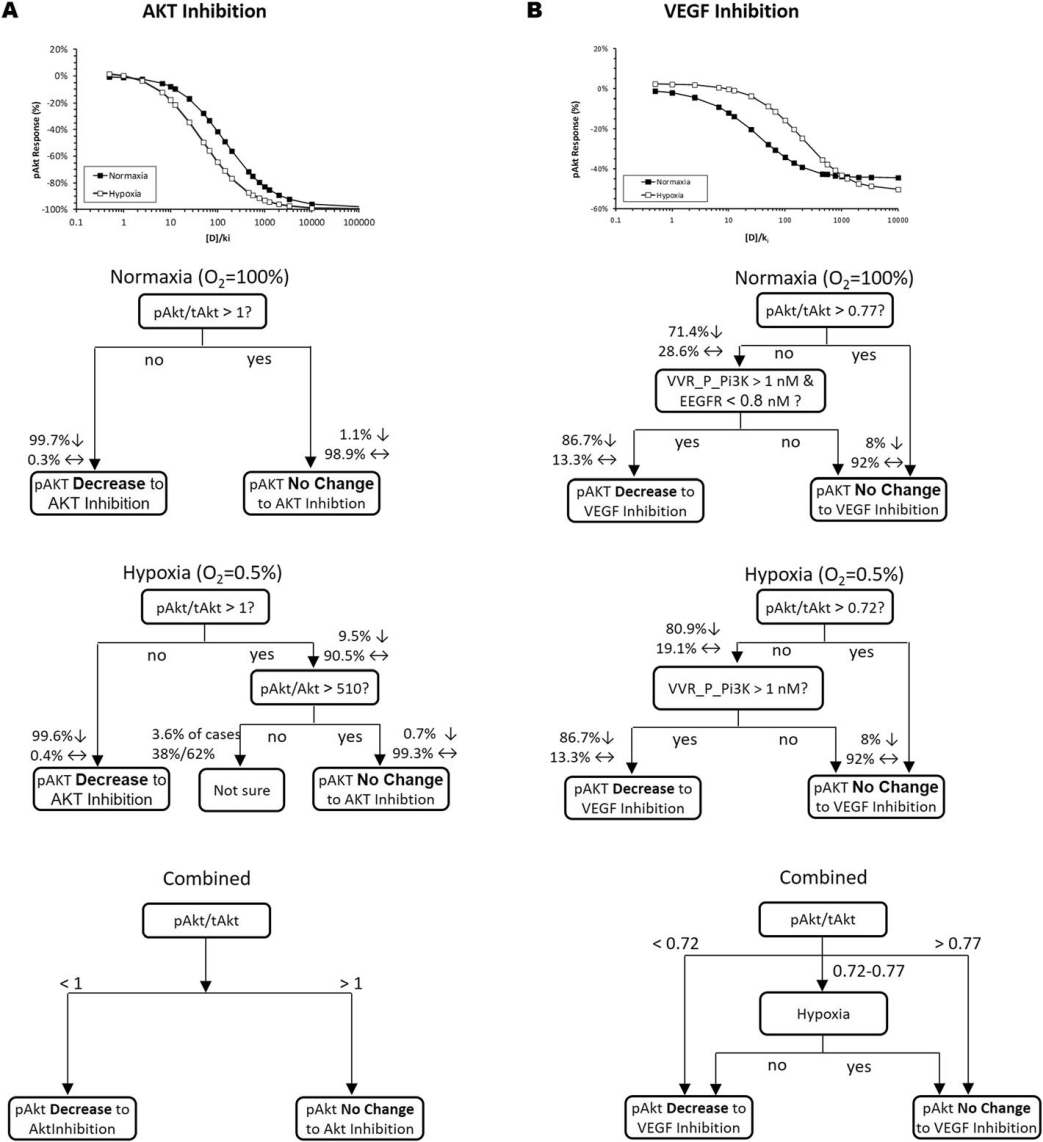
Hypoxia enhances Akt inhibitors to reduce pAkt levels, whereas the ability of VEGF inhibition to reduce pAkt is attenuated under hypoxia. Decision tree diagrams illustrating the key biomolecules dictating how pERK responses to inhibition of Akt phosphorylation (**a**) or VEGF binding (**b**). [D]/k_i_ = 200 and 1000 for inhibition of Akt phosphorylation or VEGF binding, respectively

Inhibition of Akt phosphorylation or VEGF binding to VEGFR resulted in the suppression of pAkt levels (Fig. 6). Although hypoxia enhances the effect of Akt inhibition on pAkt level, the ability of VEGF inhibition to reduce pAkt is diminished under hypoxia (Fig. 6a and b). Decision tree analysis revealed that the pAkt/tAkt ratio is the primary factor that determines if pAkt levels of the tumor cell will be sensitive to either inhibition. Tumor cells with less than complete Akt activation (pAkt/tAkt < 1) would likely be sensitive to Akt inhibition (essentially all tumor cells) (Fig. 6a). For VEGF inhibition, tumor cells with pAkt/tAkt ratio below 0.72 will be responsive regardless of O_2_ accessibility and tumor cells above 0.77 will be unresponsive. For tumor cells that fall in between the 0.72 and 0.77 pAkt/tAkt level, the sensitivity to VEGF inhibition will depend on O_2_ accessibility.

## Discussion

The rapid growth of solid tumors often leads to highly hypoxic regions with poor blood circulation. This can result in hypoxia-driven signaling that changes tumor growth dynamics and anti-cancer drug efficacy. Additionally, the growing tumor imposes a physical barrier that limits drug penetration towards the tumor core. To explore hypoxia driven tumor signaling, we developed a theoretical model by building upon published models, one that describes hypoxia signaling and the other that describes tumor growth signaling. We validated our combined mathematical model by replicating divergent hypoxia-induced Akt and ERK responses reported in literature. Additionally, by integrating ATP signaling to the model, we provide a mechanistic insight on how varying degrees of hypoxia affects current anti-cancer drugs. Our model suggests that the effect of hypoxia on ERK and Akt activation, two biomarkers of tumor proliferation and survival, and on the effectiveness of kinase and VEGF inhibitors is complex and highly dependent on the intracellular biomolecule concentrations. Using random number generation to simulate cell-to-cell variability in biomolecule concentrations followed by decision tree analysis, we identified several biomolecules whose intracellular concentration can potentially dictate the behavior of the cells.

### Effect of hypoxia on ERK and Akt activation

Experimental reports of hypoxia-induced effect on ERK and Akt activation varies considerably. For example, hypoxia (5% O_2_) was shown to decrease ERK activation [17], while a similar degree of hypoxia (7% O_2_) was shown to induce transient activation [14]. Mild hypoxia (30% O_2_) can either have no effect or cause an increase in ERK activation depending on the duration of exposure [17]. Similar divergence was also found in the effect of hypoxia on Akt activation, where it was reported to either have no effect or an increase in Akt phosphorylation [14–16].

Our analysis suggests that the differences in biomolecule concentrations among tumor cells can be an explanation for the divergent literature results. For instance, the pAkt/tAkt ratio in tumor cells in human cancer patients is highly variable, ranging from below 0.2 to higher than 0.8 [18, 19]. Our model predicts that tumor cells with pAkt/tAkt above 0.6–0.7 are likely to be unresponsive to hypoxia (Fig. 4c). For tumor cells with pAkt/tAkt below 0.6, concentrations of pPik3, EEGFR and VVR_P_PI3K become important in deciding whether the Akt activation will increase or decrease by hypoxia.

In contrast, our model suggests that ERK activation is likely to decrease with hypoxia in most cases because the criterion for “no change” (pERK/tERK < 0.9, Fig. 3c) is above those found in most tumor cells [18]. However, it is possible hypoxia can stimulate other growth factor signaling pathways that may induce an increase in ERK activity, a connection not included in our current model. With the new connection, it is feasible that there would be scenarios where hypoxia can increase or decrease ERK activity like Akt.

### Effectiveness of drug treatments

The present model provides a framework for predicting which types of tumor cells are more susceptible to anti-cancer drug treatments. For example, our analysis suggests that whether ERK activation is responsive to RAF or MEK inhibition by pharmaceutical agents depends on the intra-tumor concentration of pRAF (Fig. 5d) or pMEK (Fig. 5e). With the highly variable pRAF and pMEK levels in human tumor cells centered around 5 nM and 500 nM, respectively [20, 21], drugs acting through RAF and MEK inhibitions will be effective only in the cells with relatively low activation.

For drugs acting through inhibition of VEGF binding, the deciding factor is the pAkt/tAkt ratio (Fig. 6b). Our model predicts that tumor cells with pAkt/tAkt above 0.7 will not respond to VEGF inhibition. For cells with ratio below 0.7, the concentration of VVR_P_PI3K and EEGFR become important in deciding the effectiveness of VEGF inhibition. In contrast, the model predicts that any cells whose Akt is not completely phosphorylated (pAkt/tAkt< 1), which includes most if not all cells, will be responsive to drugs acting via Akt inhibition (Fig. 6a).

Our model also provides a framework for predicting the effectiveness of anti-cancer drugs in the hypoxic regions of tumors. Cytotoxic anti-cancer drugs whose activity depends on cell cycle progression are often less effective in hypoxic regions of tumors with reduced blood flow, not only because of the drug delivery limitation to these regions, but also because hypoxia can induce cell cycle arrest (quiescence) which protects them from these types of drugs [12, 22, 23].

However, for drugs acting through ERK, MEK or Akt inhibitions, our model predicts an augmented effect on ERK and Akt activation in hypoxic tumor regions. If drug action is not dependent on cell cycle progression (e.g. apoptosis), it is conceivable that hypoxia-induced increase in drug efficacy could partially counteract the reduced drug penetration in these hypoxic tumor regions. In contrast, our model predicts the effect of VEGF inhibiting drugs in the hypoxic regions is attenuated, thereby amplifying the already decreased effectiveness due to diminished drug reaching these regions.

### Future directions

The current model can be expanded to add additional signaling pathways such as ERK1 ability to directly phosphorylate HIF-1α [24], PI3K/Akt-induced stimulation of HIF-1α protein synthesis [25], platelet derived growth factor stimulation of glutamatergic receptors that result in the activation of ERK and Akt [26, 27], other HIF-1**α** independent pathways [28], or the effect of acidic pH levels in a solid tumor microenvironment [29].

While adding these pathways into the model could potentially modify the findings summarized above, the decision-tree based model analysis employed here may find general utility in the analysis of complex mathematical models. Uncertainty in parameters could influence the behavior of complex models that result in multiple signaling states or switch-like behavior [30]. Decision-tree based model approaches can be a powerful tool that can be used together with the more common sensitivity analysis to elucidate key behavior patterns in the models.

The role of hypoxia in combination therapy is a topic of interest in our lab and is currently on going. Our ability to successfully predict how several types of tumor cells within the tumor landscape respond to different drug-action modalities will be critical in identifying optimal targets, dosing regimen, and potential combo-therapies to achieve maximal therapeutic benefit.

## Conclusions

The interplay between hypoxic response signaling pathways and tumor signaling pathways is known to be important in the progression of cancer and the efficacy of anti-cancer therapies. We present a mathematical model that integrates hypoxia-inducible factor signaling with a tumor signaling network to explore how hypoxia and cellular heterogeneity can influence the effect of kinase or VEGF binding inhibitors. To do this we first validated our model by showing divergent response types can be generated under hypoxia using varying intracellular biomolecular concentrations. Using decision tree analysis on our model output, we were able to identify biomolecules that could be responsible for this phenomenon. Furthermore, our model provides a mechanism to explain why different cells under varying degree of hypoxia can impact drug efficacy. Overall, our modeling approach provides a framework to identify types of tumor cells that could be susceptible to anti-cancer drug treatments.

The trends revealed by our simulation study suggests a direction for future in-vitro experimental analysis, where quantifying concentrations of specific biomolecules under different hypoxic levels should be included as part of the investigative process. If verified, this would further encourage the practice of quantifying biomolecule concentrations in tumor biopsies from patients as part of the strategy toward individualized medicine. If our simulation study is not verified, then the mathematical models can be improved with the new data. In this context, the approach outlined in this work offers a hypothesis testing paradigm, rather than a tool for prediction.

## Methods

### Model structure

**Fig. 7:**
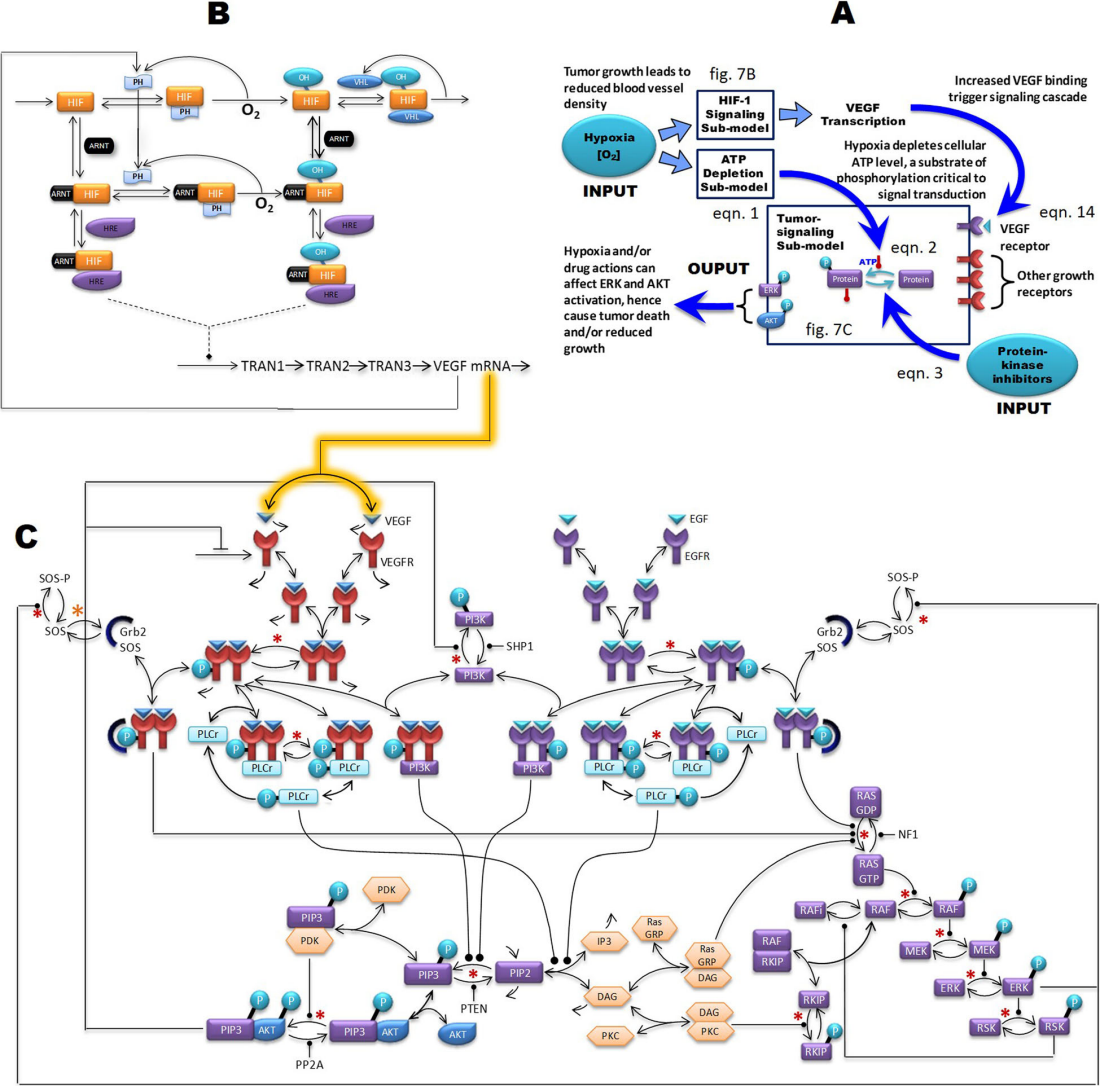
The model structure consists of HIF-1α signaling, tumor signaling, and ATP depletion modules. **a** Summary block diagram of the input, output, and the interaction between core sub-system components. Inputs: hypoxia, drug inhibition of protein kinases and inhibition of VEGF binding. Outputs: ERK and Akt activation. **b** The kinetic model of the HIF-1α signaling pathway. **c** The kinetic model of VEG and other growth factor signaling network. Stimulation and inhibition are represented as ─●and ─┤, respectively. Single arrow head toward the species denotes synthesis. The two sub-models were connected by using VEGF mRNA and the output of the HIF-1α signaling pathway model as input to the tumor signaling pathway model to drive VEGF production (arrows with yellow shadow). The hypoxia induced ATP depletion model was linked to the tumor signaling model via additions of ATP-dependent phosphorylation reactions (denoted as *). See Additional files 1 and 2 for more details

The model (Fig. 7a) consists of three modules: the HIF signaling module (Fig. 7b), the tumor signaling module (Fig. 7c), and the ATP depletion module. Both HIF and tumor signaling modulse were adopted from already published works that were integrated along with the ATP depletion module into the full model. With this full model, we examine how two hypoxia-induced mechanisms, listed below, regulate tumor signaling.

1. Hypoxia triggers HIF-1α signaling, resulting in an increase in VEGF production and VEGF receptor binding, triggering the subsequent signaling cascade through the tumor signaling network.
2. Sufficient hypoxia will cause cellular ATP depletion and thereby affects the ATP-dependent phosphorylation of signaling proteins. ATP depletion is assumed to have negligible effect on protein synthesis over the range of hypoxia considered [31].

The model inputs are: (a) level of O_2_ (% of normoxia), which can be reduced (hypoxia) due to reduced blood supply to tumors, and (b) drugs that inhibit various points of the signal transduction pathways. The model outputs are activation of ERK and Akt, chosen for their critical roles in tumor proliferation and survival.

Reactions in the model are described by set of ordinary differential equations derived from mass action laws and enzyme kinetics. The model contains 189 biomolecule species, 86 reactions and 163 parameters (see Additional files 2 and 3 for more details).

### The hypoxia-inducible factor signaling module

The hypoxia signaling module was adapted from a published work by Kohn et al. [32] that showed how reduced oxygen levels can induce a switch-like behavior of rapid increase in HIF-1α transcription factors, which is consistent with reported observation (Jewell et al. 2001, Induction of HIF-1 α in response to hypoxia). Since it has been shown that HIF-1α increases VEGF expression [8], we expanded the model to include the increased VEGF production as the result of hypoxia-induced up-regulation of HIF-1 α.

#### Tumor signaling module

To describe VEGFR signaling in the tumor, we incorporated a published model by Zhang et al. [33] to our full model. Zhang et al. model was validated by reproducing ERK and Akt dynamics in response to VEGF stimulation [33]. We further expanded the model by Zhang et al. to include signaling by endothelial growth factor (EGF), ATP-dependent reactions involving phospholipase C **γ**1(PLCR) and deactivation of Son of Sevenless (SOS) by pERK [34, 35].

#### Hypoxia induced ATP-depletion module

ATP levels only drop under severe hypoxia as cells attempt to maintain ATP homeostasis by activating compensatory changes [36, 37]. We approximated this nonlinear relationship between ATP and O_2_ using the Michaelis–Menten equation with a maximal ATP concentration of 3000 μM [38] under normoxia and a half-maximal occurring at 3.3% O_2_ [37].

1 $$ \left[ ATP\right]=\frac{{\left[ ATP\right]}_{normaxia}\left[{O}_2\right]}{0.033{\left[{O}_2\right]}_{normaxia}+\left[{O}_2\right]} $$

#### Modeling the effect of hypoxia on ATP-dependent phosphorylation

Protein phosphorylation requires binding of both ATP and protein substrates [10]. In concordance to reactions described in literature [39–41], all phosphorylation reactions follow a sequential order kinetic mechanism by which ATP binds to the enzyme first and protein substrate binding is dependent on the ATP binding [42]:

$$ Reaction\ Rate=\frac{V_{max}\left[ ATP\right]\left[ Protein\right]}{K_{M, ATP}{K}^{AB}+{K}^{AB}\left[ ATP\right]+\left[ ATP\right]\left[ Protein\right]} $$

2 $$ \kern0.5em =\frac{V_{max}\left[ Protein\right]}{\left(\frac{K_{M, ATP}}{\left[ ATP\right]}+1\right){K}^{AB}+\left[ Protein\right]} $$

*V*_*max*_ *is the maximal reaction rate, [ATP]*, and *[Protein]* are the cellular concentration of ATP and the protein substrate (e.g. RAF, MEK or Akt), respectively. *K*_*M, ATP*_ and *K*^*AB*^ are the dissociation constant of ATP with the enzyme and the ATP-enzyme complex with its protein substrate, respectively. However, Zhang et al. described their reaction without including ATP as part of the equation (Eq. 3).

3 $$ Reaction\ Rate=\frac{V_{max}^{\hbox{'}}\left[ Protein\right]}{K^{\prime }+\left[ Protein\right]} $$

Instead, the reaction rate is solely dependent on the substrate concentration. We modified Eq. 3 to explicitly include ATP as part of the ATP-dependent phosphorylation reaction in Eq. 2. To do keep *K*^′^ in Eq. 3 unchanged under normoxia, we calculated *K*^*AB*^ for each protein such that $\left(\frac{{\boldsymbol{K}}_{\boldsymbol{M},\boldsymbol{ATP}}}{{\left[\boldsymbol{ATP}\right]}_{\boldsymbol{normaxia}}}+1\right){\boldsymbol{K}}^{\boldsymbol{AB}}$ matches *K*^′^ value from the original paper, using ***K***_***M***, ***ATP***_ of 100 nM based on values reported in Knight et al. 2005 [43] for a range of proteins, and the normoxic ATP level reported in the literature [38].

#### Interfacing the HIF and tumor signaling models

The generic variable mRNA is the output of the HIF model, which represents any one of many mRNAs affected by hypoxia. In our work, we used mRNA to signify VEGF production to link HIF and tumor signaling modules. VEGF in the Zhang et al. model is synthesized at a constant rate because hypoxia induced changes in VEGF production was not considered in their original model. We modified VEGF production rate in the tumor signaling model to allow modulation of VEGF mRNA from the HIF model, thus connecting the two model components (Eq. 4).

4 $$ {k}_{syn}\left( integrated\ model\right)={k}_{syn}(original)\cdot {\left(\frac{\left[ mRNA\right]}{\left[{mRNA}_0\right]}\right)}^{k_{\alpha }} $$

The [*mRNA*] and [*mRNA*_0_] are from the original HIF model, where [*mRNA*_0_] is the mRNA concentration under normoxia. The exponent *k*_*α*_ was set to equal to 2 to account for cases where protein expression is greater than the respective mRNA level. This equation makes certain that the production of VEGF is unchanged from the value found in the original publication under normoxia. We also defined the degradation rate of VEGF protein such that the baseline VEGF level is kept equal to the value from the original paper under normoxia. Both steps ensure that the model behaves as intended in the original publication under normoxia.

### Model validation

**Fig. 8:**
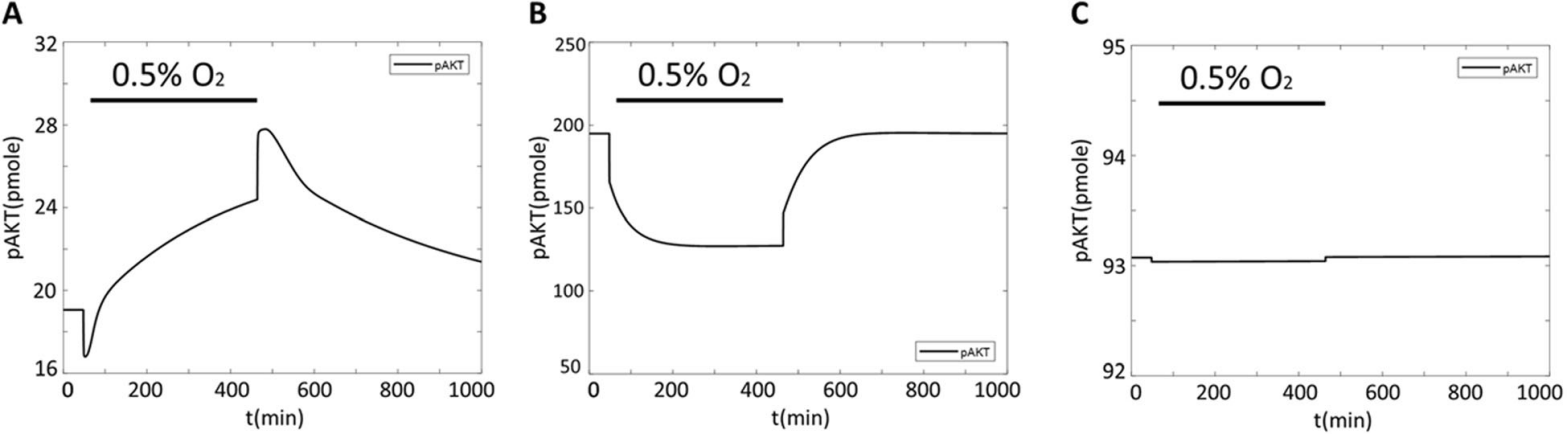
Individual timecourse simulation of pAkt response under hypoxia. Simulation examples that result in an increase (**a**), decrease (**b**), or no change (**c**) in pAKT response upon reduction in O_2_ levels as identified by horizontal bar over plot

Although our model was developed by integrating two separate published models that were validated individually, we qualitatively validated the combined full model by demonstrating the ability of our model to capture the considerable variability of hypoxia-induced effects on ERK and Akt reported in literature. There is evidence that the activation of the Akt pathway under hypoxia is cell-type specific. For example, no change in Akt response was observed in human hepatoma cells, whereas PC12 and HeLa cell-lines showed robust Akt phosphorylation under hypoxic conditions [15, 16]. Thus, we checked if our model can generate categorically dissimilar hypoxia-induced Akt responses by using different initial biomolecule values from a range that have been reported in literature (to represent different cell types where expression of these biomolecules can be very different). Indeed, our model was able to capture diverging Akt phosphorylation levels that can increase, decrease, or remain unchanged (Fig. 8) depending on initial biomolecule values under hypoxia.

### Drug actions

The four types of drug action considered in the model are: the inhibition of RAF, MEK and Akt phosphorylation and inhibition of VEGF binding to VEGFR. Since the kinase inhibitors on the market or currently in the clinic are mostly directed against the ATP-binding site of the kinase [2], we modeled inhibition of phosphorylation reactions by modifying Eq. (2) as below,

5 $$ Reaction\ Rate=\frac{V_{max}\left[ ATP\right]\left[ Protein\right]}{K_{M, ATP}{K}^{AB}\left(1+\frac{\left[I\right]}{K_i}\right)+{K}^{AB}\left[ ATP\right]+\left[ ATP\right]\left[ Protein\right]} $$

*[I]* is the concentration of drugs that inhibit RAF, MEK or Akt, and *K*_*i*_ is the inhibition constant.

For inhibition of VEGF binding to VEGFR,

6 $$ Reaction\ Rate=\frac{k_{1f}\left[ VEGF\right]\cdot \left[ VEGF R\right]}{\left(1+\frac{\left[I\right]}{K_i}\right)}-{k}_{1r}\left[ VVEGFR\right] $$

*[VEGF]*, *[VEGFR]* and *[VVEGFR]* are the concentration of VEGF, VEGF receptor, and the VEGF-VEGF receptor complex, respectively.

### Modeling and analysis approaches

We next proceeded to identify conditions that dictate the differences in cellular responses from the same stimuli and how varying degrees of hypoxia can change tumor growth and drug efficacy (see Additional file 1 for more details). Specifically:

1. How do various levels of hypoxia affect the activation of Akt and ERK?
2. How do various levels of hypoxia affect the sensitivity of Akt and ERK activation to inhibition of phosphorylation of RAF, MEK and Akt and VEGF binding by drugs?

These questions cannot be adequately addressed by evaluating the model prediction from a single set of biomolecular concentrations. Several reports have revealed cell-to-cell variability in biomolecular concentrations occur and that some even exhibit a bimodal distribution [11–13]. Furthermore, depending on the concentration of certain biomolecules, the cells may respond categorically differently to external stimuli [13]. One could view the variability in the biomolecule concentrations as subpopulations of tumor cells, each with a characteristic range of biomolecule concentrations and each could respond categorically differently from one another to the same stimuli.

In this context, a meaningful understanding of the model prediction to drug treatment under varying degrees of hypoxia requires us to (1) identify all the diverse ways the system could respond to the same stimuli because of variations in biomolecular concentrations and (2) determine which characteristics of biomolecular concentrations are responsible for inducing a deviation in the network response. To do this, we carried out the following protocol (additional details in Additional file 1):

1. For each simulation, we randomly varied the initial concentration of every biomolecules in the tumor signaling module. Latin Hypercube Sampling was used to generate evenly logarithmically spaced concentration levels over a 100-fold range to ensure coverage of the range reported in literature [11–13, 18–21]. We ran a total of 20,000 simulations, because our exploratory analysis indicated that 20,000 repetitions were sufficient to ensure statistically reproducible results. Each simulation reached steady-state before external stimuli such as changes in oxygen levels and/or addition of different drugs were introduced.
2. The simulated results of Akt and ERK activation were then categorized based on the type of response (an increase, a decrease, or no change) induced by the external stimuli.
3. For each response category, respective sets of initial biomolecule concentrations were analyzed with the decision tree algorithm [44] to identify biomolecule(s) that most likely caused the actual response.

The present study only explored the effect of variability in the tumor signaling module on the behavior of the overall model while holding the variables in other modules constant.

## Supplementary information

**Additional file 1.** Model description and simulation. This document contains additional information on model development, simulation, and analysis.

**Additional file 2: Table S2a.** Initial values for species in the tumor signaling module [45, 46]. A table of species and initial concentrations for the tumor signaling module. **Table S2b.** Initial values for species in the hypoxia signaling module. A table of species and initial concentrations for the hypoxia signaling module.

**Additional file 3: Table S3a.** Reactions for tumor signaling module. The reactions, rate equations and parameters for the tumor signaling module. Unless otherwise specified, the reactions and rate constants were taken or modified from Zhang et al. [33], Kholodenko et al. [34], Sasagawa et al. [35]. Additions by the authors are superscripted with a, b, or c, respectively. **Table S3b.** Reactions for hypoxia signaling module. The reactions, rate equations and parameters for the hypoxia signaling module. The reactions and rate constants were taken or modified from Kohn et al. [32].

## Abbreviations

(p) Akt: (phosphorylated) protein kinase B
(p) ERK: (phosphorylated) extracellular signal-regulated kinase
(p) MEK: (phosphorylated) mitogen-activated protein kinase kinase
(p) PI3K: (phosphorylated) phosphoinositide-4,5-bisphosphate 3-kinase
(p) RAF: (phosphorylated) serine/threonine-specific protein kinase
(p) Rsk: (phosphorylated) ribosomal s6 kinase
ATP: Adenosine triphosphate
EEGFR: EGF and its receptor complex
HIF: Hypoxia-inducible-factor
PIP3: Phosphatidylinositol-4,5-bisphosphate 3-kinase
PIP3_Akt: PIP3 and Akt complex
Pip3_PDK: PIP3 and phosphoinositide dependent protein kinase complex
PLCR: Phospholipase Cγ1
Rafi: Inhibited RAF
SOS: Son of Sevenless
VEGF: Vascular endothelial growth factor
VEGFR: VEGF receptor
VVR_P_PI3K: PI3K and phosphorylated dimer of VVEGFRP complex
