# Supplementary material for: Mathematical model of hypoxia and tumor signaling interplay reveals the importance of hypoxia and cell-to-cell variability in tumor growth inhibition
Source: BMC Bioinformatics. 2019 Oct 21;20:507. doi: 10.1186/s12859-019-3098-5 (PMC6802183; doi:10.1186/s12859-019-3098-5)
Supplement: Supplementary file 1 — Additional file 1. Model description and simulation. This document contains additional information on model development, simulation, and analysis. [file 12859_2019_3098_MOESM1_ESM.docx]

Additional file 1: model methods

Model Description

**The kinetic model of the HIF-1 signaling pathway.** HIFα (includes the HIF-1α and/or HIF-2α subunits of the transcription factor) and ARNT (HIF-1β) bind to form a heterodimeric transcription factor, which in turn binds the hypoxia-regulated elements (HREs) to activate the promoters under its control. The level of the heterodimeric transcription factor is regulated by proline hydroxylases (PHD s) that hydroxylate both HIFα and the heterodimeric transcription factor in an oxygen-dependent manner. Hydroxylated HIFα is thereby targeted by HIFα for degradation via binding to the von Hippel-Lindau protein (VHL), a component of ubiquitin-dependent degradation pathway. Under normaxia, the hydroxylation keeps the level of the heterodimeric transcription factor low. Under hypoxia, reduced hydroxylation leads to an increase in the level of the heterodimeric transcription factor and subsequently the production of VEGF.

**The kinetic model of VEG and other growth factor signaling network.** The tumor signaling module was based primarily on the vascular endothelial growth factor receptor (VEGFR) signaling through both the Ras-Raf-MEK-ERK and the Akt pathways developed by Zhang et al. [15], expanded to include signaling through endothelial growth factor receptor (EGFR) [16]. The signaling through the Ras-Raf-MEK-ERK pathway is initiated by the binding of GRB2 to the guanine nucleotide exchange factor SOS and the subsequent docking of the GRB2-SOS complex to phosphorylated endothelial and vascular endothelial growths factor receptors. The model also incorporated the ERK-dependent negative feedback phosphorylation of SOS, leading to the dissociation of SOS from the complex with Grb2 as described by Sasagawa *et al.* [17], as well as the ERK-mediated Raf feedback inactivation as described by Zhang *et al.* [15]. For the Akt pathways, the model captured the PI3K catalyzed formation of PIP3 from PIP2 that subsequently leads to Akt activation as well as the PLCγ catalyzed formation of inositol triphosphate (IP3) and diacylglycerol (DAG) from PIP2. The latter reaction reduces the available PIP2 to form PIP3 and hence reduces Akt activation, but the formation of IP3 and DAG also can lead to activation of the Ras-Raf-MEK-ERK pathway by (a) phosphorylation and inactivation of raf kinase inhibitor protein that inhibits ERK pathway signaling, and (b) activation of RasGRPs (a class of guanine exchange factors) that activate the Ras signaling pathway. The model also included the Akt-mediated PI3K down regulation, Akt-induced decreased receptor tyrosine kinase synthesis rates, ATP-dependent reactions involving PLCγ and deactivation of SOS by pERK [16,17].

**Incorporation of ATP into the phorsphorylation reactions.** The phosphorylation reactions in the published models [15-17] that the present work is based on were modeled according to Michaelis–Menten kinetics equation without explicitly account for the effect of changing ATP concentration due to hypoxia.

$$\boldsymbol{Reaction Rate=}\frac{\boldsymbol{V}_{\boldsymbol{max}}\left[ \boldsymbol{Protein} \right]}{\boldsymbol{K}_{\boldsymbol{M}}\boldsymbol{+}\left[ \boldsymbol{Protein} \right]}\begin{matrix} & & \boldsymbol{(S}\boldsymbol{1}a\boldsymbol{)} \end{matrix}$$

Protein phosphorylation, however, requires binding of both ATP and protein substrates to the enzyme [10]. In the present work, we modeled the phosphorylation reactions to follow a sequential order kinetic mechanism by which ATP binds to the enzyme first and protein substrate binding is dependent on the ATP binding [25]:

$$\boldsymbol{Reaction Rate=}\frac{\boldsymbol{V}_{\boldsymbol{max}}\left[ \boldsymbol{ATP} \right]\left[ \boldsymbol{Protein} \right]}{\boldsymbol{K}_{\boldsymbol{M,ATP}}\boldsymbol{K}^{\boldsymbol{AB}}\boldsymbol{+}\boldsymbol{K}^{\boldsymbol{AB}}\left[ \boldsymbol{ATP} \right]\boldsymbol{+}\left[ \boldsymbol{ATP} \right]\left[ \boldsymbol{Protein} \right]}\begin{matrix} \boldsymbol{=}\frac{\boldsymbol{V}_{\boldsymbol{max}}\left[ \boldsymbol{Protein} \right]}{\left( \frac{\boldsymbol{K}_{\boldsymbol{M,ATP}}}{\left[ \boldsymbol{ATP} \right]}\boldsymbol{+1} \right)\boldsymbol{K}^{\boldsymbol{AB}}\boldsymbol{+}\left[ \boldsymbol{Protein} \right]} & & \boldsymbol{(}S1b\boldsymbol{)} \end{matrix}$$

where *V_max_* is the maximal reaction rate, *[ATP]*, and *[Protein]* are the cellular concentration of ATP and the protein substrate (e.g. RAF, MEK or AKT), respectively. *K_M, ATP_* and *K^AB^* are the dissociation constant of ATP with the enzyme and the ATP-enzyme complex with its protein substrate, respectively.

By comparing the two equations above, it can be seen that the kinetic constant *K_M_* in *eqn.* *S1a* is a function of [ATP] that varies with the state of hypoxia.

$$\boldsymbol{K}_{\boldsymbol{M}}\boldsymbol{=}\left( \frac{\boldsymbol{K}_{\boldsymbol{M,ATP}}}{\left[ \boldsymbol{ATP} \right]}\boldsymbol{+1} \right)\boldsymbol{K}^{\boldsymbol{AB}}\begin{matrix} & & \boldsymbol{(}S1c\boldsymbol{)} \end{matrix}$$

The values of *K_M_* used in the published models therefore represent the value of the function evaluated at the [ATP] level found under normaxia.

For our model, the *K^AB^* values for each phosphorylation reaction were calculated from the corresponding *K_M_* values used in the published models [15,16] using *eqn. S1c* above. For the calculations, 3000 μM was used as the [ATP] at normaxia^21^ and *K_M,ATP_* = 100 μM based on published data for 110 kinases [44].

Model Simulation

SimBiology (version 5.0, The MathWorks, Natick, MA) was used to construct the model. MATLAB (R2014a, The MathWorks, Natick, MA) was used for all numerical simulations. The MATLAB variable-order differential equation solver ode15s based on the numerical differentiation formulas was configured with the following parameters: absolute tolerance, 1e^-6^, the relative tolerance, 1e^-3^.

Latin Hypercube Sampling. Latin hypercube sampling was used to generate a matrix of random initial values. The initial levels of the biomolecules were assumed to be distributed uniformly with a 100-fold range of the values from other experiments or literatures. The algorithm shuffles the sample for each input so that there is no correlation between the inputs. The MATLAB Latin hypercube sampling function call *lhsdesign* was configured with the following parameters: criterion, ‘correlation’, number of iterations, 25.

Decision Tree Algorithm. The classification and regression trees algorithm (CART) developed by Breiman *et al*. [30] was used to construct decision trees using the repeated simulations as training set. The construction of each decision tree begins by the separation of the simulations into categories according to its response to external stimuli (e.g. does the pAkt level increase or decrease with hypoxia). The algorithm then asks a sequence of hierarchical Boolean questions about how best to split the biomolecule concentrations (e.g. pPI3K>20 nM) in order to best partition the training sets according to their response categories. The default algorithm constructs the full decision tree first and then prunes it to yield subtrees that do not overfit the training data. The smallest subtree that is within one standard error of the minimum cost subtree is regarded as the tree pruned to the best level. The MATLAB *fitctree* function implementing the CART algorithm was configured with the following parameters: maximum number of categories, 10, maximal number of branches, number of cases - 1, minimum number of observations at each leaf, 50. The accuracy of trees is evaluated with the MATLAB *predict* function to estimate the accuracy of trees.
