# Supplementary material for: Mathematical model of hypoxia and tumor signaling interplay reveals the importance of hypoxia and cell-to-cell variability in tumor growth inhibition
Source: BMC Bioinformatics. 2019 Oct 21;20:507. doi: 10.1186/s12859-019-3098-5 (PMC6802183; doi:10.1186/s12859-019-3098-5)
Supplement: Supplementary file 2 — Additional file 2: Table S2a. Initial values for species in the tumor signaling module [45, 46]. A table of species and initial concentrations for the tumor signaling module. Table S2b. Initial values for species in the hypoxia signaling module. A table of species and initial concentrations for the hypoxia signaling module. [file 12859_2019_3098_MOESM2_ESM.docx]

**Additional file 2: model species and values**

**Table 2a.** Initial values for specie in the hypoxia signaling module. A table of species and initial concentration for the hypoxia signaling module.

| **Index** | **Abbreviation** | **Descriptions** | **Initial value**  **(pmol/mL)** |
| --- | --- | --- | --- |
| 1 | VEGFR | receptors for vascular endothelial growth factor | 200 |
| 2 | VEGF | vascular endothelial growth factor | 0.02^[45,46]^ |
| 3 | VVEGFR | the complex of VEGF and VEGFR | 0 |
| 4 | VVEGFR2 | dimer of the complex of VEGF and VEGFR | 0 |
| 5 | VVEGFRP | phosphorylated dimer of the complex of VEGF and VEGFR | 0 |
| 6 | PLCR | phospholipase C γ1 | 250 |
| 7 | PLCRP | phosphorylated PLCR | 0 ^a^ |
| 8 | PI3K | Phosphatidylinositol-4,5-bisphosphate 3-kinase | 200 |
| 9 | PI3KP | phosphorylated PI3K | 0 |
| 10 | PIP3 | phosphatidylinositol (3,4,5)-trisphosphate | 0 |
| 11 | PIP2 | phosphatidylinositol 4,5-bisphosphate | 1000 |
| 12 | SOS | Son of Sevenless; Ras-guanine exchange factor | 34 ^a^ |
| 13 | SOSP | phosphorylated SOS | 0 ^a^ |
| 14 | GRB2 | growth factor receptor-bound protein 2 | 85 ^a^ |
| 15 | GRB2_SOS | complex of GRB2 and SOS | 82.3 |
| 16 | VVR_P_PI3K | complex of VVEGFRP and PI3K | 0 |
| 17 | VVR_P_PLCR | complex of VVEGFRP and PLCR | 0 |
| 18 | VVR_P_PLCRP | phosphorylated VVR_P_PLCR | 0 |
| 19 | VVR_P_GS | complex of VVEGFRP and GRB2_SOS | 0 |
| 20* | PDK | phosphoinositide-dependent protein kinase | 100 |
| 21 | Akt | protein kinase B | 100 |
| 22 | PIP3_PDK | complex of PIP3 and PDK | 0 |
| 23 | PIP3_Akt | complex of PIP3 and Akt | 0 |
| 24 | PIP3_AktP | phosphorylated PIP3-Akt | 0 |
| 25 | PKC | protein kinase C | 200 |
| 26 | DAG | diacylglycerol | 0 |
| 27 | DAG_PKC | complex of DAG and PKC | 0 |
| 28 | RAF | serine/threonine-specific protein kinases | 20 |
| 29 | RKIP | RAF kinase inhibitor protein | 20 |
| 30 | RKIP_RAF | complex of RKIP and RAF | 0 |
| 31 | RKIPP | phosphorylated RKIP | 0 |
| 32 | RAFP | phosphorylated RAF | 0 |
| 33 | RAFI | inhibited RAF | 0 |
| 34 | RSK | ribosomal s6 kinase | 100 |
| 35 | RSKP | phosphorylated RSK | 0 |
| 36 | ERK | extracellular signal-regulated kinases | 400 |
| 37 | ERKP | phosphorylated ERK | 0 |
| 38 | MEK | mitogen-activated protein kinase kinase | 200 |
| 39 | MEKP | phosphorylated MEK | 0 |
| 40 | RASGTP | RAS bound to guanosine triphosphate | 0 |
| 41 | RASGDP | RAS bound to guanosine diphosphate | 95.7 |
| 42 | RASGRP | RAS guanyl nucleotide-releasing protein | 82.3 |
| 43 | DAG_RASGRP | the complex of DAG and RASGRP | 0 |
| 44 | IP3 | inositol 1,4,5-trisphosphate | 0 |
| 45* | PTEN | phosphatase and tensin homolog | 100 |
| 46* | SHP1 | Src homology region 2 domain-containing phosphatase-1 | 100 |
| 47* | NF1 | Neurofibromin 1 | 25 |
| 48* | PP2A | protein phosphatase 2A | 20 |
| 49 | EGF | endothelial growth factor | 1^a^ |
| 50 | EGFR | endothelial growth factor receptor | 100 ^a^ |
| 51 | EEGFR | complex of EGF and EGFR | 0 ^a^ |
| 52 | EEGFR2 | dimer of the complex of EGF and EGFR | 0 ^a^ |
| 53 | EEGFRP | phosphorylated EEGFR2 | 0 ^a^ |
| 54 | EER_P_PI3K | complex of EEGFRP and PI3K | 0 ^a^ |
| 55 | EER_P_PLCR | complex of EEGFRP and PLCR | 0 ^a^ |
| 56 | EER_P_GS | complex of EEGFRP and GRB2_SOS | 0 ^a^ |
| 57 | EER_P_PLCRP | phosphorylated EER_P_PLCR | 0 ^a^ |

**Notes:**

1. The initial values for species superscripted with a were taken from Kholodenko *et al.* [34]. The initial values for all other species were taken from Zhang *et al.* [33].
2. The concentration of the species superscripted with * were kept constant during simulations.

Table 2b. Initial values for specie in the hypoxia signaling module. A table of species and initial concentration for the hypoxia signaling module.

| **Index** | **Abbreviation** | **Descriptions** | **Initial value**  **(pmol/mL)** |
| --- | --- | --- | --- |
| 58 | OXYGEN | oxygen | 1  (normaxia) |
| 59 | HIFPRE | precursor of HIF | 1 |
| 60 | DegradationP | degraded protein | 0 |
| 61 | HIF | hypoxia-inducible factor (HIF-1α) | 5 |
| 62 | ARNT | HIF-1β | 5 |
| 63 | HIF_ARNT | complex of HIF and ARNT | 0 |
| 64 | HRE | hypoxia-responsive elements | 1 |
| 65 | HIF_ARNT_HRE | complex of HIF and ARNT and HRE | 0 |
| 66 | TRANI1 | transition before TRAN2 | 0 |
| 67 | TRANI2 | transition before TRAN3 | 0 |
| 68 | TRANI3 | transition before mRNA | 0 |
| 69 | MRNA | mRNA | 0 |
| 70 | PH | proline hydroxylases | 10 |
| 71 | HIF_PH | complex of HIF and PH | 0 |
| 72 | HIFOH | hydroxylated HIF | 0 |
| 73 | HIF_ARNT_PH | complex of HIF, ARNT and PH | 0 |
| 74 | HIFOH_ARNT | complex of HIFOH and ARNT | 0 |
| 75 | VHL | von Hippel-Lindau protein | 10 |
| 76 | HIFOH_VHL | complex of HIFOH and VHL | 0 |
| 77 | HIFOH_ARNT_VHL | complex of HIFOH, ARNT and VHL | 0 |
| 78 | HIF_ARNT_HRE_PH | complex of HIF, ARNT and HRE, PH | 0 |
| 79 | HIFOH_ARNT_HRE | complex of HIFOH, ARNT and HRE | 0 |

- The initial values for species in this table were taken from Kohn *et al.* [32]
