# Supplementary material for: Mathematical model of hypoxia and tumor signaling interplay reveals the importance of hypoxia and cell-to-cell variability in tumor growth inhibition
Source: BMC Bioinformatics. 2019 Oct 21;20:507. doi: 10.1186/s12859-019-3098-5 (PMC6802183; doi:10.1186/s12859-019-3098-5)
Supplement: Supplementary file 3 — Additional file 3: Table S3a. Reactions for tumor signaling module. The reactions, rate equations and parameters for the tumor signaling module. Unless otherwise specified, the reactions and rate constants were taken or modified from Zhang et al. [33], Kholodenko et al. [34], Sasagawa et al. [35]. Additions by the authors are superscripted with a, b, or c, respectively. Table S3b. Reactions for hypoxia signaling module. The reactions, rate equations and parameters for the hypoxia signaling module. The reactions and rate constants were taken or modified from Kohn et al. [32]. [file 12859_2019_3098_MOESM3_ESM.docx]

**Additional file 3: model reactions**

**Table 3a. Reactions for tumor signaling module.** The reactions, rate equations and parameters for the tumor signaling module. Unless otherwise specified, the reactions and rate constants were taken or modified from Zhang et al. [33], Kholodenko et al. [34], Sasagawa et al. [35] or added by the authors are superscripted with a, b, or c, respectively.

| **No.** | **Reactions** | **Descriptions and parameter values** |
| --- | --- | --- |
| 1 | VEGF + VEGFR ⮀ VVEGFR  *V_1f_* = k1f* VEGF* VEGFR/(1+Drug /ki,VEGF); *V_1r_* = k1r* VVEGFR | VEGF binding to VEGF Receptor, modified to account for inhibition of binding by drug  K1f=0.48 ml/pmol/min; k1r=0.06 min^-1^ |
| 2 | VVEGFR + VVEGFR ⮀ VVEGFR2  *V_2f_* = k2f* VVEGFR* VVEGFR; *V_2r_* = k2r* VVEGFR2 | VEGF_VEGFR dimerization  K2f=0.036 ml/pmol/min; k2r=0.06 min^-1^ |
| 3 | VVEGFR2 ⮀ VVEGFRP  *V_3f_* = k3f* VVEGFR2*(1+ks3/ATP0)/(1+ks3/ ATP) ; *V_3r_* = k3r* VVEGFRP | Autophosphorylation of VEGF_VEGFR dimer, modified to account for ATP concentration  K3f=60 ml/pmol/min; k3r=0.6 min^-1^;ks3=1e^5^ pmol/ml |
| 4 | VEGFR → *ϕ*  *V_4f_* = k4d* VEGFR | VEGFR degradation  k4d=0.01 min^-1^ |
| 5 | VVEGFR → *ϕ*  *V_5f_* = k5d* VVEGFR | VEGF_VEGFR degradation  k5d=0.01 min^-1^ |
| 6 | VVEGFRP → *ϕ*  *V_6f_* = k6d* VVEGFRP | (VEGF_VEGFR)P degradation  k6d=0.1 min^-1^ |
| 7 | VVEGFR2 → *ϕ*  *V_7f_* = k7d* VVEGFR2 | VEGF_VEGFR dimer degradation  k7d=0.01 min^-1^ |
| *8* | *ϕ* → VEGFR  *V_8f_* = ksyn*(1-( PIP3_AKTP)^kn^/(kh^kn+( PIP3_AKTP)^kn^)) | VEGFR production, which is inhibited by AktP  ksyn=1.7 pmol/ml/min; kn=3 |
| *9^c^* | *ϕ* ⮀ VEGF  *V_9f_* = kin_v *( mRNA/mRNA0^k_alpha^ ; *V_9r_* = kout_v* VEGF | VEGF production (regulated by mRNA output from the hypoxia signaling module) and degradation  k_alpha=2; kout_v=1.7 ml/min;  kin_v= (kout_v*vegf0 + k1f*vegf0*vegfrs-k1r*vvegfrs) |
| 10 | PLCR + VVEGFRP ⮀ VVR_P_PLCR  *V_10f_* = k10f* PLCR* VVEGFRP; *V_10r_* = k10r* VVR_P_PLCR | PLCr binding to (VEGF_VEGFR)P  k10f=0.091 ml/pmol/min; k10r=6 min^-1^ |
| 11^a^ | VVR_P_PLCR⮀VVR_P_PLCRP  *V_11f_* = k11f* VVR_P_PLCR*(1+ks11/ATP0)/(1+ks11/ ATP) ; *V_11r_* = k11r* VVR_P_PLCRP | phosphorylation of VVRP_PLCR  k11f=60 ml/pmol/min; k10r=3 min^-1^; ks11=1e^5^ pmol/ml |
| 12^a^ | VVR_P_PLCRP ⮀ VVEGFRP + PLCRP  *V_12f_* = k12f* VVR_P_PLCRP; *V_12r_* = k12r* VVEGFRP* PLCRP | dissociation of PLCRp from the dimer VVRP_PLCRP  k12f=18 ml/pmol/min; k12r=0.36 min^-1^ |
| 13 | PI3K + VVEGFRP ⮀ VVR_P_PI3K  *V_13f_* = k13f* PI3K* VVEGFRP; *V_13r_* = k13r* VVR_P_PI3K | PI3K binding to VVEGFRP  k13f=0.84 ml/pmol/min; k13r=0.2 min^-1^ |
| 14 | GRB2_SOS + VVEGFRP ⮀ VVR_P_GS  *V_14f_* = k14f* GRB2_SOS* VVEGFRP; *V_14r_* = k14r* VVR_P_GS | GRB2_SOS binding to VVEGFRP  k14f=0.06 ml/pmol/min; k14r=2.7 min^-1^ |
| 15 | RASGDP + DAG_RASGRP + VVR_P_GS → RASGTP + VVR_P_GS + DAG_RASGRP  *V_15f_* = k15*( VVR_P_GS+ DAG_RASGRP)* RASGDP/((ks15*kab15/ ATP+kab15)+ RASGDP) | Phosphorylation of RASGDP, modified to account for ATP concentration  k15=15 min^-1^; ks15=1e^5^ pmol/ml; kab15=193.55 pmol/ml |
| 16 | PIP2 + VVR_P_PI3K → VVR_P_PI3K + PIP3  *V_16f_* = k16* VVR_P_PI3K* PIP2/((ks16*kab16/ ATP+kab16)+ PIP2) | Phosphorylation of PIP2, modified to account for ATP concentration  k16=750 min^-1^; ks16=1e^5^ pmol/ml; kab16=1103.2 pmol/ml |
| 17^a^ | EGF + EGFR ⮀ EEGFR  *V_17f_* = k17f* EGF* EGFR; *V_17r_* = k17r* EEGFR | EGF binding to EGFR  k17f=0.18 ml/pmol/min; k17r=3.6 min^-1^ |
| 18^a^ | EEGFR + EEGFR ⮀ EEGFR2  *V_18f_* = k18f* EEGFR* EEGFR; *V_18r_* = k18r* EEGFR2 | EGF_EGFR forms dimer, from 1999  K18f=0.6 ml/pmol/min; k18r=6 min^-1^ |
| 19^a^ | EEGFR2 ⮀ EEGFRP  *V_19f_* = k19f* EEGFR2*(1+ks19/ATP0)/(1+ks19/ ATP) ; *V_19r_* = k19r* EEGFRP | Autophosphorylation of EGF_EGFR dimer, modified to account for ATP concentration  K19f=60 ml/pmol/min; k19r=0. 6 min^-1^ |
| 20^a^ | GRB2_SOS + EEGFRP ⮀ EER_P_GS  *V_20f_* = k20f* GRB2_SOS* EEGFRP; *V_20r_* = k20r* EER_P_GS | GRB2_SOS binding to EEGFRP  k20f=0.06 ml/pmol/min; k20r=2.7 min^-1^ |
| 21 | RASGDP + EER_P_GS → RASGTP + EER_P_GS  *V_21f_* = k15* EER_P_GS* RASGDP/((ks15*kab15/ ATP+kab15)+ RASGDP) | Phosphorylation of RASGDP, modified to account for ATP concentration  k15=15 min^-1^; ks15=1e^5^ pmol/ml; kab15=193.6 pmol/ml |
| 22^a^ | EEGFRP + PLCR ⮀ EER_P_PLCR  *V_22f_* = k21f* PLCR* EEGFRP; *V_22r_* = k21r* EER_P_PLCR | PLCR binding to EEGFRP  k21f=3. 6 ml/pmol/min; k21r=12 min^-1^ |
| 23^a^ | EEGFRP + PI3K ⮀ EER_P_PI3K  *V_23f_* = k22f* PI3K* EEGFRP; *V_23r_* = k22r* EER_P_PI3K | PI3K binding to EEGFRP  k22f=0.84 ml/pmol/min; k22r=0.2 min^-1^ |
| 24 | PIP2 + EER_P_PI3K → EER_P_PI3K + PIP3  *V_24f_* = k16* EER_P_PI3K* PIP2/((ks16*kab16/ ATP+kab16)+ PIP2) | Phosphorylation of PIP2, modified to account for ATP concentration  k16=750 min^-1^; ks16=1e^5^ pmol/ml; kab16=1103.23 pmol/ml |
| 25^a^ | EER_P_PLCR ⮀ EER_P_PLCRP  *V_25f_* = k23f* EER_P_PLCR*(1+ks23/ATP0)/(1+ks23/ ATP) ; *V_25r_* = k23r* EER_P_PLCRP | Phosphorylation of EERP_PLCR, modified to account for ATP concentration  k23f=60 ml/pmol/min;k23r=3 min^-1^; ks23=1e^5^ pmol/ml |
| 26^a^ | EER_P_PLCRP ⮀EEGFRP + PLCRP  *V_26f_* = k24f* EER_P_PLCRP; *V_26r_* = k24r* EEGFRP* PLCRP | PLCRP dissociation from EERP_PLCRP  k24f=18 ml/pmol/min; k24r=0.36 min^-1^ |
| 27^a^ | PLCRP → PLCR  *V_27f_* = v25* PLCRP/( PLCRP+km25) | Phosphorylation of PLCRP  v25=60 pmol/ml/min; km25=100 pmol/ml |
| 28^b^ | SOS + ERKP → SOSP + ERKP  *V_28f_* = k26* SOS* ERKP/((kab26*ks26/ ATP+kab26)+ SOS) | Phosphorylation of SOS, modified to account for ATP concentration  k26=60 min^-1^; ks26=1e^5^ pmol/ml; kab26=24814.8 pmol/ml |
| 29^b^ | SOSP → SOS  *V_29f_* = k27* SOSP | SOSP dephosphorylated  k27=0.00012 min^-1^ |
| 30^a^ | GRB2 + SOS ⮀ GRB2_SOS  *V_30f_* = k28f* GRB2* SOS; *V_30r_* = k28r* GRB2_SOS | GRB2 binding to SOS  k28f=0.006 ml/pmol/min; k28r=0.09 min^-1^ |
| 31 | PIP2 + PLCRP → DAG + IP3 + PLCRP  *V_31f_* = k29* PIP2* PLCRP/(km29+ PIP2) | Formation of DAG and IP3 from PIP2  k29=0.12 min^-1^; km29=600 pmol/ml/min |
| 32 | PI3KP + SHP1 → PI3K + SHP1  *V_32f_* = k30* SHP1* PI3KP/(km30+ PI3KP) | PI3KP dephosphorylation  k30=36 min^-1^; km30=630 pmol/ml/min |
| 33 | PIP3 + PTEN → PIP2 + PTEN  *V_33f_* = k31* PTEN* PIP3/(km31+ PIP3) | PIP3 dephosphorlyation  k31=300 min^-1^; km31=1100 pmol/ml/min |
| 34 | PIP3 + PDK ⮀ PIP3_PDK + PDK  *V_34f_* = k32f* PIP3* PDK; *V_34r_* = k32r* PIP3_PDK | PIP3 binding to PDK (PDK is assumed to remain constant)  k32f=0.18 ml/pmol/min; k32r=60 min^-1^ |
| 35 | PIP3 + AKT ⮀ PIP3_AKT  *V_35f_* = k33f* PIP3* AKT; *V_35r_* = k33r* PIP3_AKT | PIP3 binding to Akt  k33f=18 ml/pmol/min; k33r=60 min^-1^ |
| 36 | PIP3_AKT + PIP3_PDK → PIP3_AKTP + PIP3_PDK  *V_36f_* = k34* PIP3_PDK* PIP3_AKT/((ks34*kab34/ ATP*(1+ drug_akt/ki_akt)+kab34)+ PIP3_AKT) | PIP3_AKT phosphorylation, modified for ATP as well as inhibition by drug  k34=180 ml/pmol/min; ks34=1e^5^ pmol/ml; kab34=96.8 pmol/ml |
| 37 | PIP3_AKTP + PP2A → PIP3_AKT + PP2A  *V_37f_* = k35* PP2A* PIP3_AKTP/(km35+ PIP3_AKTP) | PIP3_AKTP dephosphorylation  k35=90 min^-1^; km35=100 pmol/ml/min |
| 38 | DAG + PKC ⮀ DAG_PKC  *V_38f_* = k36f* DAG*cell.PK; *V_38r_* = -k36r* DAG_PKC | DAG binding to PKC  k36f=0.11 ml/pmol/min; k36r=6 min^-1^ |
| 39 | DAG + RASGRP ⮀ DAG_RASGRP  *V_39f_* = k37f* DAG* RASGRP; *V_39r_* = k37r* DAG_RASGRP | DAG binding to RASGRP  k37f=0.1 ml/pmol/min; k37r=0.05 min^-1^ |
| 40 | RKIP + RAF ⮀ RKIP_RAF  *V_40f_* = k38f* RKIP* RAF; *V_40r_* = k38r* RKIP_RAF | RKIP binding to RAF  k38f=18 ml/pmol/min; k38r=0.3 min^-1^ |
| 41 | RKIP + DAG_PKC → RKIPP + DAG_PKC  *V_41f_* = k39* DAG_PKC* RKIP/((ks39*kab39/ ATP+kab39)+ RKIP) | RKIP phosphorylation, modifeid with ATP concentration, original  k39=180 min^-1^; ks39=1e^5^ pmol/ml; kab39=290.3 pmol/ml |
| 42 | RKIPP → RKIP  *V_42f_* = v40* RKIPP/(km40+ RKIPP) | RKIPP dephosphorylation  v40=60 pmol/ml/min; km40=99 pmol/ml/min |
| 43 | RAF + RASGTP → RAFP + RASGTP  *V_43f_* = k41* RASGTP* RAF/((ks41*kab41/ ATP*(1+ drug_raf/ki_raf)+kab41)+ RAF) | RAF phosphorylation, modified with ATP as well as drug inhibition  k41=27 min^-1^; ks41=1e^5^ pmol/ml; kab41=40.2 pmol/ml |
| 44 | RAFP → RAF  *V_44f_* = v42* RAFP/(km42+ RAFP) | RAFP dephosphorylation  v42=160 pmol/ml/min; km42=9.96 pmol/ml/min |
| 45 | MEK + RAFP → RAFP + MEKP  *V_45f_* = k43* MEK* RAFP/((ks43*kab43/ ATP*(1+ drug_mek/ki_mek)+kab43)+ MEK) | MEK phosphorylation, modified to account for ATP concentration  k43=50 min^-1^; ks43=1e^5^ pmol/ml; kab43=14.5 pmol/ml |
| 46 | MEKP → MEK  *V_46f_* = v44* MEKP/(km44+ MEKP) | MEKP dephosphorylation  v44=1500 pmol/ml/min; km44=996 pmol/ml/min |
| 47 | ERK + MEKP → ERKP + MEKP  *V_47f_* = k45* MEKP* ERK/((ks45*kab45/ ATP+kab45)+ ERK) | ERK phosphorylation, modified to account for ATP concentration  k45=8.3 min^-1^; ks45=1e^5^ pmol/ml; kab45=14.5 pmol/ml |
| 48 | ERKP → ERK  *V_48f_* = v46* ERKP/(km46+ ERKP) | ERKP dephosphorylation  v46=330 pmol/ml/min; km46=996 pmol/ml/min |
| 49 | RSK + ERKP → RSKP + ERKP  *V_49f_* = k47* ERKP* RSK/((ks47*kab47/ ATP+kab47)+ RSK) | RSK phosphorylatio, modified to account for ATP concentration  k47=18 min^-1^; ks47=1e^5^ pmol/ml; kab47=9.7 pmol/ml |
| 50 | RSKP → RSK  *V_50f_* = k48* RSKP | RSKP dephosphorylation  k48=18 min^-1^ |
| 51 | RASGTP + NF1 → RASGDP + NF1  *V_51f_* = k49* NF1* RASGTP/(km49+ RASGTP) | RASGTP desphosphorylation  k49=130 min^-1^; km49=260 pmol/ml/min |
| 52 | RAF + RSKP → RAFI + RSKP  *V_52f_* = k50* RSKP* RAF/(km50+ RAF) | RAF inactivated by RSKP  k50=27 min^-1^; km50=42 pmol/ml/min |
| 53 | RAFI → RAF  *V_53f_* = v51* RAFI/(km51+ RAFI) | RAFI activated  v51=160 pmol/ml/min; km51=9.96 pmol/ml/min |
| 54 | PI3K + PIP3_AKTP → PI3KP + PIP3_AKTP  *V_54f_* = k52* PIP3_AKTP* PI3K/((ks52*kab52/ ATP+kab52)+ PI3K) | PI3K phosphorylated (inactivated), , modified to account for ATP concentration  K52=2000 min^-1^; ks52=1e^5^ pmol/ml; kab52=1936 pmol/ml |
| 55 | IP3 → *ϕ*  *V_55f_* = k53d* IP3 | IP3 degradation  k53d=4.8 min^-1^ |
| 56 | DAG → *ϕ*  *V_56f_* = k54d* DAG | DAG degradation  k53d=0.065 min^-1^ |
| *57* | *ϕ* → PIP2  *V_57f_* = k55s | PIP2 production  k55s=2.9 pmol/min |
| 58 | PIP2 → *ϕ*  *V_58f_* = k56d* PIP2 | PIP2 degradation  k56d=0.0029 min^-1^ |

**Table 3b. Reactions for hypoxia signaling module.** The reactions, rate equations and parameters for the hypoxia signaling module. The reactions and rate constants were taken or modified from Kohn et al. [32] .

| **No.** | **Reactions** | **Descriptions and parameter values** |
| --- | --- | --- |
| 59 | HIFPRE → HIFPRE + HIF  *V_59f_* = k65s | HIF production  k65s = 11.58 pmol/ml/min |
| 60 | HIF → DegradationP  *V_60f_* = k66d* HIF | HIF degradation  k66d = 0.042 min^-1^ |
| 61 | HIF + ARNT ⮀ HIF_ARNT  *V_61f_* = k67f* HIF* ARNT; *V_61r_* = k67r* HIF_ARNT | HIF binds with ARNT  k67f=0.888 ml/pmol /min; k67r = 100 min^-1^ |
| 62 | HIF_ARNT + HRE ⮀ HIF_ARNT_HRE  *V_62f_* = k68f* HIF_ARNT* HRE; *V_62r_* = k68rHIF_ARNT_HRE | HIF_ARNT binds with HRE  k68f = 16 ml/pmol/min; k68r = 4.8 min^-1^ |
| 63 | HIFOH_ARNT_HRE → TRANI1 + HIFOH_ARNT_HRE  *V_63f_* = k69* HIFOH_ARNT_HRE | TRANI1 production  k69 = 2.4 min^-1^ |
| 64 | HIF_ARNT_HRE → TRANI1 + HIF_ARNT_HRE  *V_64f_* = k70* HIF_ARNT_HRE | TRANI1 production  k70 = 2.4 min^-1^ |
| 65 | TRANI1 → TRANI2  *V_65f_* = k71* TRANI1 | TRANI2 production  k71 = 2.4 min^-1^ |
| 66 | TRANI2 → TRANI3  *V_66f_* = k72* TRANI2 | TRANI3 production  k72 = 2.4 min^-1^ |
| 67 | TRANI3 → mRNA  *V_67f_* = k73* TRANI3 | mRNA production  k73 = 2.4 min^-1^ |
| 68 | mRNA → DegradationP  *V_68f_* = k74d* mRNA | mRNA degradation  k74 = 2.4 min^-1^ |
| 69 | HIF + PH ⮀ HIF_PH  *V_69f_* = k75f* HIF* PH; *V_69r_* = k75r* HIF_PH | HIF binding to PH  K75f = 93 ml/pmol/min; k75r= 2.5 min^-1^ |
| 70 | HIF_PH + OXYGEN → PH + HIFOH + OXYGEN  *V_70f_* = k76* HIF_PH* OXYGEN | HIF gets Ohized  K76 = 1.4 ml/pmol /min |
| 71 | HIF_ARNT + PH ⮀ HIF_ARNT_PH  *V_71f_* = k77f* HIF_ARNT* PH; *V_71r_* = k77r* HIF_ARNT_PH | HIF_ARNT binding to PH  K77f = 93 ml/pmol/ min; k77r = 2.5 min^-1^ |
| 72 | HIF_ARNT_PH + OXYGEN → PH + HIFOH_ARNT + OXYGEN  *V_72f_* = k78* HIF_ARNT_PH* OXYGEN | HIF_ARNT_PH gets Oxidized  K78 = 1.4 ml/pmol/min |
| 73 | HIFOH + VHL ⮀ HIFOH_VHL  *V_73f_* = k79f* HIFOH* VHL; *V_73r_* = -k79r* HIFOH_VHL | HIFOH binding to VHL  k79f=28.4 ml/pmol/ min; k79r= 8.3 min^-1^ |
| 74 | HIFOH_VHL → VHL  *V_74f_* = k80* HIFOH_VHL | HIFOH degradation  k80 = 12.8 min^-1^ |
| 75 | ARNT + HIFOH ⮀ HIFOH_ARNT  *V_75f_* = k81f* ARNT* HIFOH; *V_75r_* = k81r* HIFOH_ARNT | HIFOH binding to ARNT  k81f = 0.88 ml/pmol/ min; k79r = 100 min^-1^ |
| 76 | HRE + HIFOH_ARNT ⮀ HIFOH_ARNT_HRE  *V_76f_* = k82f* HRE* HIFOH_ARNT; *V_76r_* = k82r* HIFOH_ARNT_HRE | HRE binding to HIFOH_ARNT  k82f = 16 ml/pmol/ min; k82r = 4.85 min^-1^ |
| 77 | mRNA → PH + mRNA  *V_77f_* = k83*mRNA | PH production  k83 = 0.024 min^-1^ |
| 78 | PH → DegradationP  *V_78f_* = k84* PH | PH degradation  k84 = 0.06 min^-1^ |
